# Shape and rate of movement of the invasion front of *Xylella fastidiosa* spp. *pauca* in Puglia

**DOI:** 10.1038/s41598-020-79279-x

**Published:** 2021-01-13

**Authors:** David Kottelenberg, Lia Hemerik, Maria Saponari, Wopke van der Werf

**Affiliations:** 1 Wageningen University, Biometris, P.O. Box 16, 6700 AA Wageningen, The Netherlands; 2 Consiglio Nazionale delle Ricerche, Istituto per la Protezione Sostenibile Delle Piante, Bari, via Amendola 122/D, Bari, Italy; 3 Wageningen University and Research, Centre for Crop Systems Analysis, P.O. Box 430, 6700 AA Wageningen, The Netherlands

**Keywords:** Plant sciences, Biological models, Ecology, Ecological modelling

## Abstract

In 2013, *Xylella fastidiosa* spp. *pauca* was first reported in Puglia, Italy, causing the olive quick decline syndrome (OQDS). Since then the disease has spread, prompting the initiation of management measures to contain the outbreak. Estimates of the shape of the disease front and the rate of area expansion are needed to inform management, e.g. the delineation of buffer zones. However, empirical estimates of the invasion front and the rate of spread of OQDS are not available. Here, we analysed the hundreds of thousands of records of monitoring data on disease occurrence in Puglia to estimate the shape of the invasion front and the rate of movement of the front. The robustness of estimation was checked using simulation. The shape of the front was best fitted by a logistic function while using a beta-binomial error distribution to model variability around the expected proportion of infected trees. The estimated rate of movement of the front was 10.0 km per year (95% confidence interval: 7.5–12.5 km per year). This rate of movement is at the upper limit of previous expert judgements. The shape of the front was flatter than expected. The fitted model indicates that the disease spread started approximately in 2008. This analysis underpins projections of further disease spread and the need for preparedness in areas that are still disease free.

## Introduction

There is a crisis in Puglia, southern Italy, due to a new plant disease caused by bacterium *Xylella fastidiosa* spp. *pauca*. The bacterium causes a disease that is named olive quick decline syndrome (OQDS)^1^.

It is estimated that 8,000 hectares of olive orchards were affected by the bacterium in 2013, the year of first reporting of the disease^1–3^. In December 2014, one year after first report, the known infected area had almost tripled^4^. OQDS severely impacts the local economy of the Puglia region as the symptoms progress rapidly on the trees, which become unproductive and eventually die. In affected areas, production and movement of plants for planting is regulated, causing a decline in income from nursery production^5,6^.

Olives are symbolic trees for the region and the olive orchards are part of the local landscape and cultural heritage^6^. The loss of often centuries-old trees greatly affects the cultural heritage and attractiveness of the region. Farms have been the property and livelihood of families for many years and generations, and the locals find a lot of pride in their farms. Although these consequences of the invasion are hard to monetize, the impacts are large.

**Figure 1:**
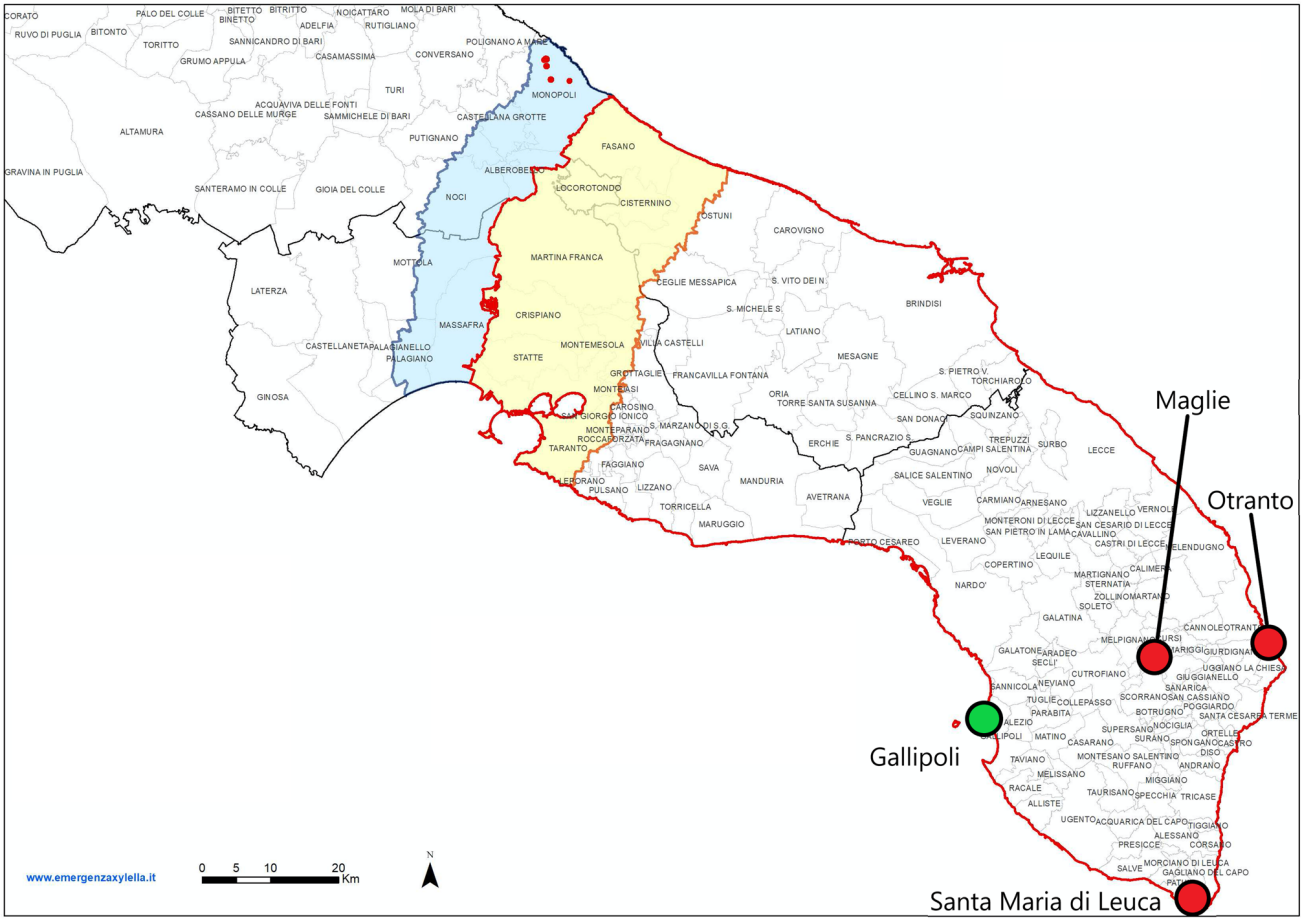
Demarcated areas in October 2020 in the Puglia region, southern Italy. The red outlined region is the whole infected zone. Within the infected zone, the yellow region is the containment zone, where all infected plants must be removed, and all host plants within a 100 m radius of the infected plants are tested. The blue region is the buffer zone, where every infected plant found should be removed, as well as all host plants within a 100 m radius of the infected plant. The red dots in the blue region are newly found infected plants in the buffer zone for which the demarcated zones have not been updated yet. The green dot is the town of Gallipoli, the presumed origin of the outbreak of *X. fastidiosa* in Puglia. The red dots are the towns of Maglie, Otranto, and Santa Maria di Leuca. These locations are used as alternative origins of the outbreak in sensitivity analyses on the point of origin. The image was obtained from https://www.emergenzaxylella.it.

**Figure 2:**
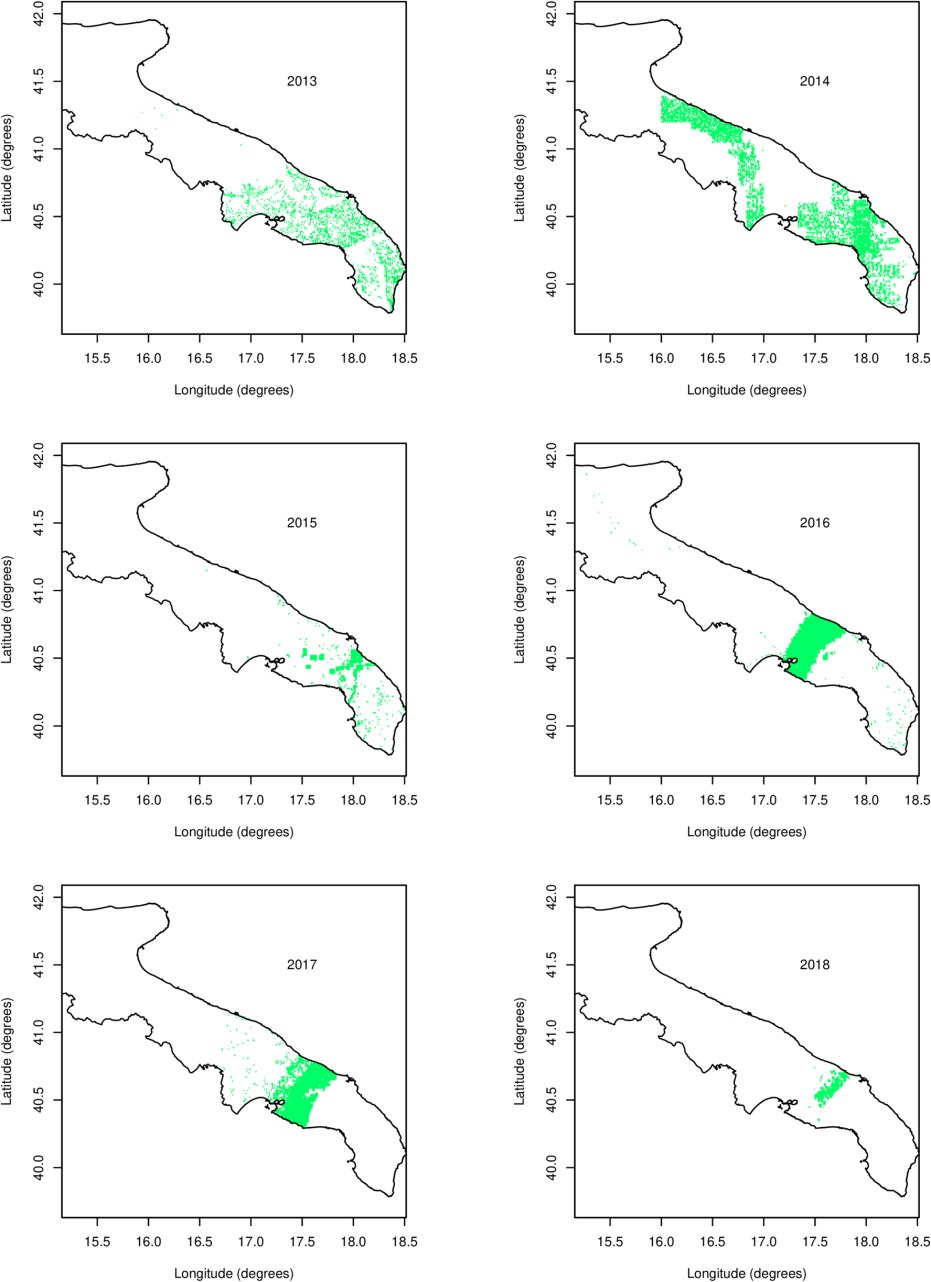
The sampling pattern in the Puglia region per year, 2013–2018. Green spots are locations where samples have been taken. Sampling was done by the Apulian Regional Phytosanitary Service. Latitude and Longitude are expressed in degrees.

**Figure 3:**
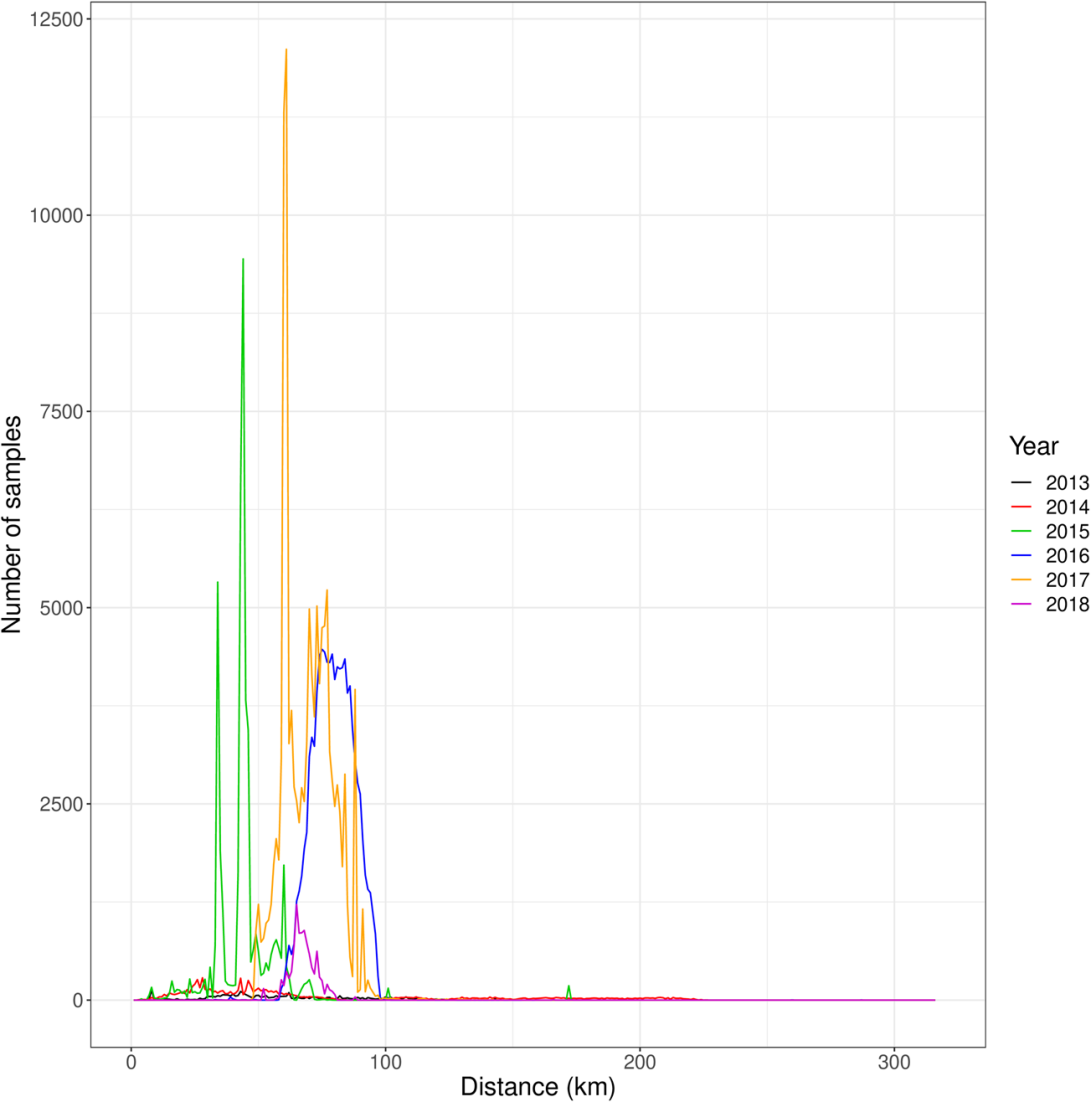
Sampling intensity in the Puglia region. The number of samples is given for each 1 km-wide ring as a function of distance to the town of Gallipoli, the presumed origin of the outbreak.

A campaign to monitor disease spread and intercept new advanced foci has been in place in Puglia since November 2013. Samples have been taken from olive trees and other known susceptible host species to assess the presence of *X. fastidiosa*. The sampling strategy is based on the delineation of three so-called “demarcated areas”^7^: (1) the infected zone, which is the area that contains all known infected trees; (2) the containment zone, which is the 20 km wide northern part of the infected zone where infections are regularly found and specific control operations are mandatory; and (3) the buffer zone, which is the 10 km wide disease free area immediately outside the infected zone where strict measures are taken to prevent the disease from establishing. The demarcation of the areas is based on the prescriptions of the EU Decision 2015/789, recently amended by the EU Regulation 2020/1201, and is continuously updated in relation to the results of each monitoring campaign (Fig. 1). As the sampling follows the requirements of regulations, which have been updated through time, the sampling pattern is very heterogeneous in space (Fig. 2) and the number of samples as a function of distance to the presumed origin of the disease outbreak, Gallipoli, varies substantially from year to year (Fig. 3). Given the primary objective of the surveillance program (i.e. early detection of potential new infections at the forefront of the invasion) sampling and testing have been implemented preferentially in a specific band of the region: the buffer zone in which the bacterium is supposedly not yet present, and in the containment zone, 20 km wide, located between the heavily infected area and the buffer zone. After 2015, a very limited number of trees have been monitored in the area declared infected, and the monitored territories varied according to the continuous change in the demarcation of the areas as consequence of findings of new outbreaks. There is also a large difference in the number of samples taken each calendar year (Fig. 3). The irregularity of the sampling pattern in space, and the large differences between years in where samples were taken, make the analysis of the data challenging. The irregularity of the sampling strategy could result in a biased analysis result when using these data. However, solving the problem of estimation with such irregular data is important, because such irregularity is the rule rather than the exception in datasets of disease invasions^8–10^.

Several models have been developed to analyse and predict the spread of *X. fastidiosa*. White et al.^11^ modelled the spread of *X. fastidiosa* using a spatially explicit simulation model. The model was calibrated on the spatial data on disease presence from surveys. This model was used to make calculations on the optimal width of the buffer zone and make calculations on disease survey and detection efficiency and inform management. Soubeyrand et al.^12^ developed an SIR model (susceptible, infectious, recovered for the three compartments of the model) to describe the epidemiology of *X. fastidiosa* in southern France. They found that the introduction of *X. fastidiosa* in France could have occurred around 1985, suggesting there may be hidden compartments in which the bacterium was not detected for a long time^12^. Hidden compartments might exist for other *X. fastidiosa* introductions as well, implying that the first detection of the bacterium could be many years after its introduction. The model of Soubeyrand et al.^12^ focuses on disease progress in time and ignores spatial aspects. Strona et al.^13^ developed an epidemic network model. They found that in the orchard network in Puglia many nodes have many connections, as opposed to most other real-world networks. This implies that containment of the bacterial spread is very challenging. Finding practical solutions to cope with the infections in the heavily infected area is one of the research priorities.

Abboud et al.^14^ analysed the spread of *X. fastidiosa* in Southern-Corsica with a reaction–diffusion-absorption equation to estimate the moment of introduction of the bacterium. They found that the pathogen moves with 155 m/month, or 1.86 km per year^14^. Furthermore, they estimated that the pathogen was introduced to the area in 1959, long before its first detection in 2015^12,14^. The environment in Corsica is quite different from the environment of Puglia, meaning that the spread of disease can be different between these areas. However, these findings do emphasize that an introduction well before detection is a likely scenario, and this could very well be the case for *X. fastidiosa* in Puglia.

Several biological and epidemiological questions have recently been addressed, e.g. the pathogenicity of the introduced bacterial strain, and the importance of different insect vector species. However, two important basic ecological questions have not been answered, namely “what is the shape of the disease front”, and “what is the rate of spread of this new invasion?”. Information on the shape of the front (along a cross section parallel to the direction of spread) and the rate of spread of novel pathogens is required to inform management and define buffer and containment zones in which surveillance and eradication measures are implemented. Buffer zones could also be defined on the basis of the dispersal of the pathogen, which occurs both at short- and long-range. However, while insect vectors are primarily responsible for short range pathogen dispersal, long range jumps also contribute to the expansion of the epidemic front. Active short-range movement of the main vector, *Philaenus spumarius*, has been quantified experimentally, but it is difficult to estimate the long range movement, particularly because the vector could hitch-hike with vehicles, like tractors and trucks^15^. Hence, the long-range dispersal of vectors, as used in models, has previously not been based on rigorous data analysis, but was based on scenario assumptions^11,16^. Estimating the rate of movement of the disease front empirically could in part alleviate the difficulty of estimating the rate of spread of the disease on the basis of the movement of vectors.

The front of a spreading population is often described using an exponential or logistic equation^17,18^. The shape of the disease front of *X. fastidiosa* has not been determined. The speed at which this front moves through Puglia has also not been determined. The European Food Safety Authority EFSA organized an expert knowledge elicitation to answer the question “What is the mean distance which will comprise 90% of the area containing the newly infected plants around an infected area in 1 year?”. Based on this elicitation, EFSA estimated that 90% of newly infected plants within a year will fall within 5.2 km of a previously infected area (95% confidence limit (CL) of 0.73–14.0 km)^19^. This estimate was used in a recent assessment of the economic impact *X. fastidiosa* spp. *pauca* in Europe^20^. Up to now, there has been no actual estimation for the rate of spread of OQDS in Puglia based on analysis of available empirical data.

Here, we analyse the spread of *X. fastidiosa* in Puglia based on sampling in the region between 2013 and 2018. Various combinations of deterministic and stochastic models were fitted to the data to assess the shape of the dispersal front. Using the best fitting shapes for the shape of the front, the rate of spread (i.e. the rate of movement of the front) was estimated. Stochastic simulation was used to demonstrate that the procedure that we used to estimate the rate of spread results in unbiased estimates, even with the irregular and year-to-year varying spatial support of the sampling data. Based upon the fitted front shapes, we estimate the width of the front (from 5 to 95% diseased trees). Furthermore, by extrapolating the far tail of the moving front back to the postulated place of origin of the invasion, we estimate the year of introduction of the pathogen in Puglia.

## Materials and methods

Samples included in this dataset were taken from olive trees sampled from November 2013 until April 2018 by the Apulian Regional Phytosanitary Service. From April 2016 to April 2018, sampling was done only in the buffer zone and containment zone (Fig. 1) and was structured in quadrats of one hectares (ha) area, with at least one sample collected in each quadrat. Within each quadrat, priority was given to sample symptomatic trees and if within the quadrat several trees showed disease symptoms, these were also sampled and individually tested. Samples consisted of mature olive twigs (at least 8 twigs/tree), collected close to symptomatic branches, or from the 4 cardinal points of the canopy when sampling asymptomatic trees. The samples were first tested for *X. fastidiosa* by using Enzyme-linked immunosorbent assay (ELISA)^21^. All ELISA-positive samples, and those yielding doubtful ELISA results, plus 3% of the negative samples, were subsequently tested using quantitative PCR.

**Figure 4:**
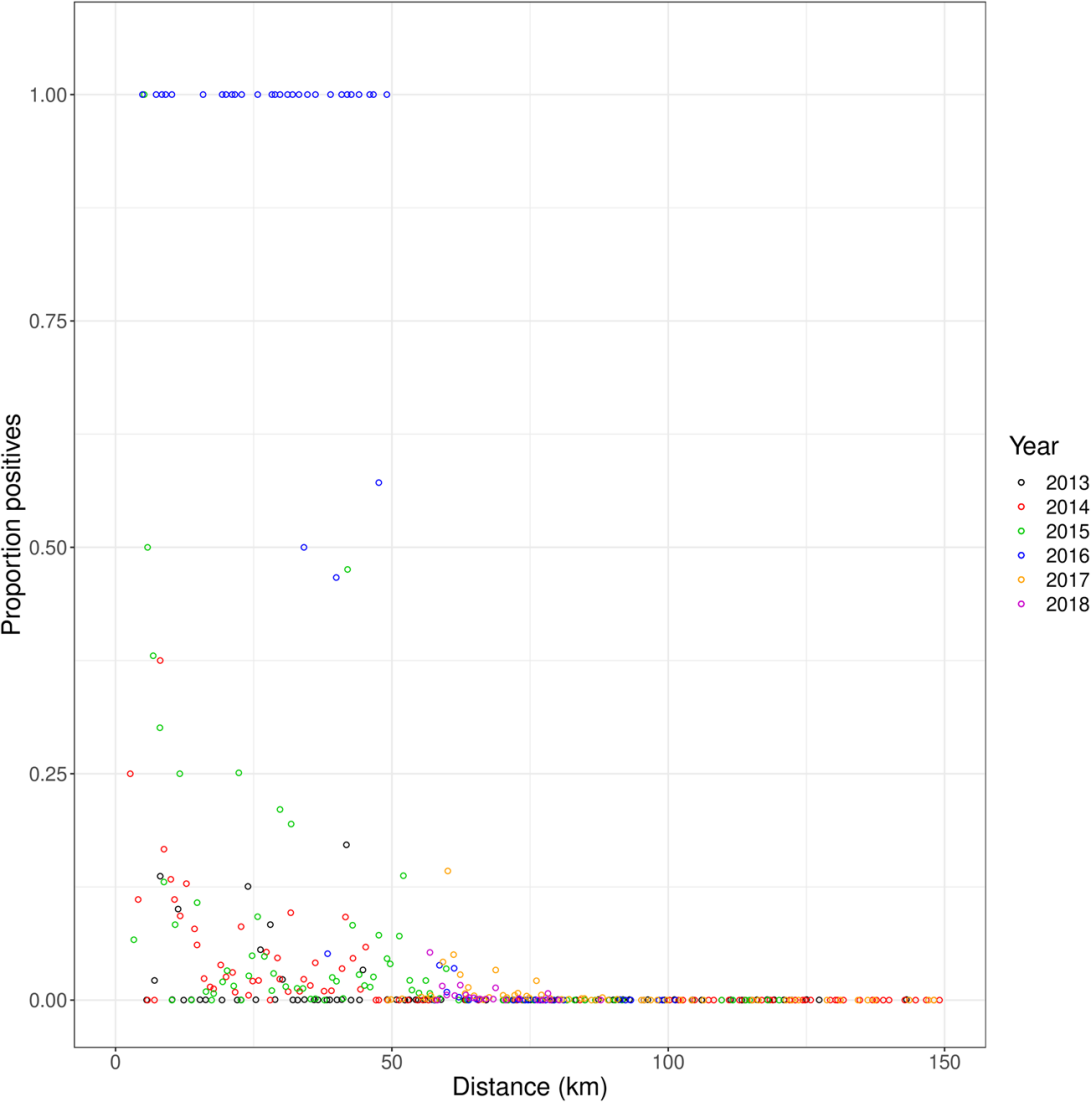
Relationship between proportion of positive samples per each km ring (Y-axis) and distance to Gallipoli (X-axis; km). Points with different colour represents different years.

The total data set comprises 409,515 records and 7 columns. The columns are the ID number of the measurement, longitude, latitude, result (0 for negative on *X. fastidiosa* presence, 1 for positive), day, year, and month. The number of rows was reduced to 298,230 rows after removing NA (not available) values for the result column or missing coordinates for the longitude and latitude columns. We initially tried to work with the point data as observed, but found that these data were extremely difficult to analyse, presumably because of large variability in the data leading to very flat likelihood surfaces that did not support convergence of the optimization algorithms tested for fitting spatial expansion models (Simplex, Simulated annealing, etc.). We therefore grouped the observation data in 1-km wide distance classes from the port of Gallipoli, the likely origin of the disease invasion (latitude: 40.055851, longitude: 17.992615)^22^ and calculated the proportion of infected trees in each class. We thus obtained a reduced data set with approximately 200 distance classes comprising an inner circle of 1 km radius, and concentric rings of 1 km width each, with for each class the number of sampled trees and the number of infected trees. We then analysed the relationship between the proportion of infected trees and the distance from Gallipoli (Fig. 4). This relationship was first identified separately for each year, and subsequently by assuming a constant rate of displacement over time (i.e. the rate of spread) of a disease front with a fixed shape.

We expected a high proportion of positive samples at short distance from Gallipoli, with the proportion declining with increasing distance. Therefore, we chose for the shape of the disease front the following deterministic functions (1) a negative exponential function, (2) a decreasing logistic function, and (3) a constrained negative exponential function (CNE; constrained to have a maximum proportion diseased trees $p = 1.0$) (Table 1). The shape of the tail of the invasion front is in many instances exponential^18,23–26^, but the proportion of disease cannot exceed one, hence the CNE was used as a modification of an exponential relationship. The sampled data is binary count data (number of positive samples out of the total number of samples at a given distance) and the distance is transformed to discrete distance circles. Because the data are based on a known number of samples in each distance class with a stochastic number of positive outcomes, we chose the binomial distribution and the beta-binomial distribution as candidate stochastic models for fitting the model to the data (Table 1). The binomial model is a model for count data with a defined maximum (*N*), assuming a fixed probability of “success” (infection). The beta-binomial takes overdispersion into account by drawing the probability of success from a beta distribution around the mean probability of success. The probability of success, i.e. the proportion of positive samples, depends on the distance from Gallipoli and the time since first detection. In our model for the invasion front, the mean probability of disease presence at a distance $x$ from Gallipoli is described by the deterministic part of the model (e.g. logistic), while the beta-binomial variability in the detection result is described by an overdispersion parameter $\theta$ which increases in value as the variance tends towards the variance of the binomial distribution (Bolker, 2008). Mathematically, the parameter *θ* equals the sum of the parameters $a + b$, where $a$ and $b$ are the shape parameters of the beta distribution^27^. Given a same mean, the beta-binomial distribution has a larger variance than the binomial distribution (Table 1). The beta-binomial distribution tends to the binomial distribution as $\theta$ gets large. For all model fits, we calculated the AIC (Akaike information criterion):

1 $${\text{AIC}} = 2k - 2 \log \left( L \right)$$

**Table 1:** Deterministic and stochastic models used for fitting all combinations of deterministic and stochastic models.

Deterministic model	Function		
Negative exponential	$f_{1} \left( x \right) = a \cdot {\text{exp}}\left( { - rx} \right)$		
Logistic	$f_{2} \left( x \right) = \frac{1}{{1 + {\text{exp}}\left( {r\left( {x - x_{50} } \right)} \right)}}$		
CNE*	$$f_{3} \left( x \right) = { }\left\{ {\begin{array}{ll} {1 \; \mid \; x < x_{100} ,} \\ {\exp \left( { - r\left( {x - \left( {x_{100} + ct} \right)} \right)} \right) \; \mid \; x \ge x_{100} .} \\ \end{array} } \right.{ }$$		

Stochastic model	function	Mean	Variance
Binomial distribution	$$g_{1} \left( {x,N,p} \right) = \left( {\begin{array}{*{20}c} N \\ x \\ \end{array} } \right)p^{x} \left( {1 - p} \right)^{N - x}$$	$Np$	$Np\left( {1 - p} \right)$
Beta-binomial distribution	$g_{2} \left( {x,N,p,\theta } \right) = \frac{\Gamma \left( \theta \right)}{{\Gamma \left( {p\theta } \right)\Gamma \left( {\left( {1 - p} \right)\theta } \right)}}\frac{N!}{{x!\left( {N - x} \right)!}}\frac{{\Gamma \left( {x + p\theta } \right)\Gamma \left( {N - x + \left( {1 - p} \right)\theta } \right)}}{{\Gamma \left( {N + \theta } \right)}}$	$Np$	$Np\left( {1 - p} \right)\left( {1 + { }\frac{N - 1}{{\theta + 1}}} \right)$

Parameters are described in the “Materials and methods” section.

*CNE* constrained negative exponential.

where $k$ is the number of estimated parameters, log is the natural logarithm, and *L* is the likelihood^27^. The model with the lowest AIC was selected as the most supported model. Models with a difference in AIC from the minimum AIC model of two or less are considered equivalent. In that case, we selected the simplest model.

Next, we used the two best fitting models (see “Results” section), the logistic function with beta-binomial distribution and the CNE function with beta-binomial distribution, to analyse the speed with which *X. fastidiosa* spreads through Puglia. To keep the models in a simplified form, it can be assumed that the dispersal front retains its shape over time and space and moves in space at a constant rate^28,29^. Therefore, for this analysis the deterministic functions from Table 1 are modified to include a yearly spread rate *c* (km per year) and time variable *t* (year):

2 $${\text{Logistic}}\;{\text{function:}}\;p_{l} = \frac{1}{{1 + {\text{exp}}\left( {r\left( {x - (x_{50} + ct} \right)} \right))}}$$

3 $${\text{CNE}}\;{\text{function:}}\;p_{c} = \left\{ {\begin{array}{ll} 1 & { \mid\; x < x_{100} + ct,} \\ {\exp \left( { - r\left( {x - \left( {x_{100} + ct} \right)} \right)} \right) } & {\mid\; x \ge x_{100} + ct.} \\ \end{array} } \right.$$

where $p_{l}$ and $p_{c}$ are the proportion of positive measurements of the logistic and CNE functions respectively, $r$ is the relative growth rate of the disease in the tail in km^-1^, $x$ is the distance in km from the disease origin, Gallipoli, $x_{50}$ is the (negative) *x*-value (distance from Gallipoli) of the half-maximum of the curve at $t = 0$ in km, $x_{100}$ is the (negative) $x$-value where the CNE function curve reaches a value of 1.0 at $t = 0$ in km, $t$ is the time since 2013 in years, and the parameter *c* is the rate of spread in km per year. With these equations, one curve for every $t$ (year) is displayed. 95% confidence limits (CLs) were calculated with the likelihood ratio test method^27^.

To test the adequacy of the methodology for estimating the shape of the invasion front and the rate of spread, we did stochastic simulations in which we generated data on an expanding disease, collected samples in the same spatially heterogeneous manner from the simulated data as we did for the actual data sets, and re-estimated the rate of spread from the data. The estimated parameter values were then compared to the known parameter input values. The simulations were done using the logistic function and CNE function for the shape of the disease front and a beta-binomial distribution to describe variability. Data was randomly generated using a beta-binomial distribution for every distance circle according to the expected proportion of disease ($p$) calculated from the deterministically moving front, while the number of samples (*N*) within each distance circle was the same as in the empirical data. Again, a constant shape and rate of spread of the dispersal front is assumed^29^. Because of the uncertainty regarding the location of the front when sampling started (2013) and the rate of spread, the parameters that describe these aspects of the model, $x_{50}$ (logistic) or $x_{100}$ (CNE) and $c$ respectively, were also varied in the stochastic simulations. For the logistic function, the parameters $r$ (the relative growth rate of the disease in the tail) and $\theta$ (overdispersion) were fixed at 0.08 km^−1^ and 1 respectively, while parameter $x_{50}$ was varied from − 40 to − 5 km from Gallipoli with steps of 5 km, and the parameter $c$ was varied from 5 to 16 km per year with steps of 1 km per year. For the CNE function, the parameters $r$ and $\theta$ were again fixed at 0.08 km^−1^ and 1 respectively, while parameter $x_{100}$ was varied from − 45 to − 10 km with steps of 5 km, and parameter $c$ was varied from 5 to 16 km per year with steps of 1 km per year. Data generation and estimation of parameters was done 10 times for each combination of parameters. For every combination of the location parameter, $x_{50}$ or $x_{100}$, and the rate of range expansion, *c*, the mean difference between the set rate of spread and the estimated rate of spread was calculated ($X_{i}$; where *i* is the index for a parameter combination). Using the generated set of differences *X*_*i*_, we calculated the mean bias ($\overline{X}$):

4 $$\overline{X} = \frac{{\mathop \sum \nolimits_{i}^{n} X_{i} }}{n}$$

where $n$ is the total number of parameter combinations. We also calculated the root-mean-squared error (RMSE):

5 $${\text{RMSE}} = \sqrt {\frac{{\mathop \sum \nolimits_{i}^{n} X_{i}^{2} }}{n}}$$

We estimated the width of the invasion front using a logistic shape of the invasion front. Width was calculated as the distance between the 1st and 99th percentile of the front or between the 5th and 95th percentile. For this, a curve at any point in time can be used since the curves have the same shape, and the width is the same in every year (Fig. 6). For the logistic function and the calculation of the 1st and 99th percentile the following applies:

6 $$\frac{1}{{1 + {\text{exp}}\left( {r\left( {x_{99} - \left( {x_{50} + ct} \right)} \right)} \right)}} = 0.99$$

7 $$\frac{1}{{1 + {\text{exp}}\left( {r\left( {x_{1} - \left( {x_{50} + ct} \right)} \right)} \right)}} = 0.01$$

This is solved to find:

8 $$x_{1} - x_{99} = \frac{{2{\text{log}}\left( {99} \right)}}{r}$$

where log is the natural logarithm. Using Eq. (7), we also estimate the supposed starting time of the logistic growth of the disease by calculating $t$ for $x_{1} = 0$.

To assess the sensitivity of our analysis to the point of origin, for which we chose Gallipoli in accordance with the best available evidence, we repeated our analyses of the shape of the front and the rate of spread when assuming different points of origin. For this we use three fictitious origin locations (Fig. 1): Santa Maria di Leuca, Otranto, and Maglie. We choose Santa Maria di Leuca and Otranto because these are also cities in Puglia with ports. We choose Maglie because it lies approximately in between the other three locations. These locations are not chosen because we think they are plausible points where *Xylella* could have been introduced for the first time, but only because they are suitable locations for a sensitivity analysis. To further asses the sensitivity of choosing Gallipoli as the point of origin, we repeat our simulations when generating data with Santa Maria di Leuca, Otranto, or Maglie as the point of origin, but analyse this data assuming Gallipoli as the point of origin.

All calculations and model fitting were done in R 3.6.0^30^. The complete dataset and details on the data analysis are available in the R script online at https://github.com/DBKottelenberg/OQDS_Xf_Puglia.

## Results

### Shape of the front

For every deterministic model (a negative exponential function, a logistic function, or a CNE function), the beta-binomial distribution was a better stochastic model than the binomial distribution (Table 2). This means that there is overdispersion in the data when grouped within the distance circles.

**Table 2:** Akaike information criterion (AIC) for the logistic and constrained exponential model when fitted to data for each year separately.

	Binomial distribution			Beta-binomial distribution		
	Negative exponential	Logistic	CNE	Negative exponential	Logistic	CNE
**Data with all years** **Year**						
2013	159	159	159	**78**	**78**	**78**
2014	344	344	344	**232**	**232**	**232**
2015	8853	8847	8847	**457**	**457**	**457**
2016	587	239	239	221	**139**	145
2017	4673	4442	4442	**334**	**334**	**334**
2018	87	87	87	108	**77**	**77**
Total	14,703	17,227	14,169	1430	**1317**	1323
Total without 2016	14,153	13,916	13,954	1209	**1177**	1178

Bold values are the lowest AIC values within a row.

*CNE* constrained negative exponential.

**Figure 5:**
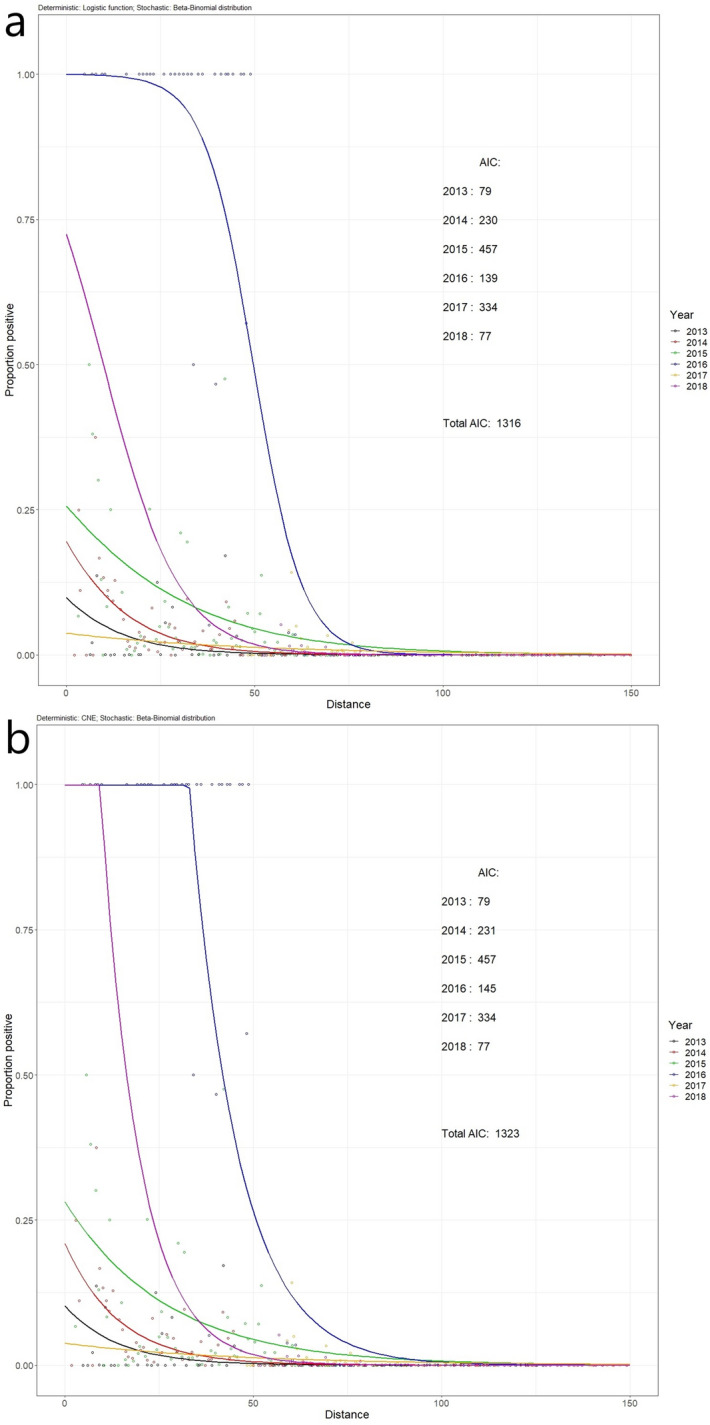
Logistic and constrained negative exponential (CNE) functions fitted to the data of each year separately. The stochastic model was a beta-binomial distribution in all cases. The AICs are noted in the graph. (**a**) Logistic function; (**b**) CNE function.

The logistic and CNE functions fitted the data equally well, except for 2016 where the logistic model fitted better (lower AIC) (Table 2 and Fig. 5). The negative exponential function fitted worse than the logistic and CNE models in each year. An overall AIC calculated by summing the year-specific AICs over the years was lowest for the logistic model, showing that, overall, the data is better fitted with a logistic function than with a CNE function. The difference in overall AIC was due to the differing AICs for 2016.

### Rate of movement of the disease front

**Figure 6:**
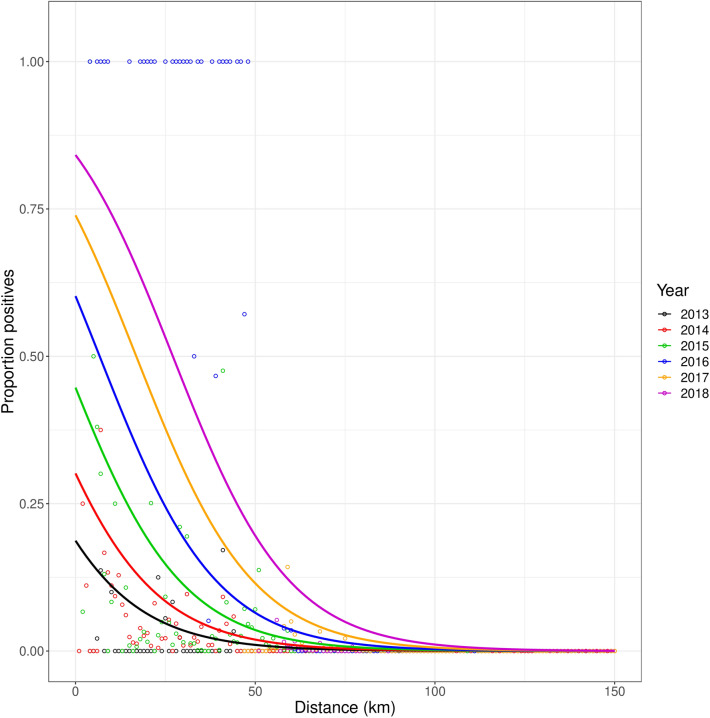
Modelled advance of the logistic front of *X. fastidiosa* at a yearly time step, 2013–2018. The disease front was fitted on all data using a logistic model containing a parameter for the rate of range expansion *c* and assuming a beta-binomial error distribution (Eq. 2).

Assuming that the shape of the front of the invasion stays the same, the rate of movement of the front was estimated for the logistic function with beta-binomial distribution and CNE function with beta-binomial distribution (Table 3). The rate of movement for these functions was estimated at 10.0 km per year (95% confidence interval (CI): 7.5–12.5 km per year) for the logistic and 10.2 km per year (95% CI: 7.7–12.6 km per year) for the CNE. Figure 6 shows the movement of the disease front over time modelled with the logistic function.

**Table 3:** Parameter estimates of the logistic and constrained negative exponential functions when fitted to data for all years, and assuming a constant rate of movement. Values in brackets are the 95% confidence limits.

Parameter (unit)	Function	
	Logistic	CNE
**Data with all years**		
*r*	0.059 km^−1^ (0.047, 0.072 km^−1^)	0.056 km^−1^ (0.044, 0.067 km^−1^)
*x*_50_/*x*_100_	− 25.76 km (− 39.46, − 15.36 km)	− 31.71 km (− 44.71, − 22.83 km)
*c*	9.95 km/year (7.48, 12.49 km/year)	10.19 km per year (7.74, 12.63 km/year)
*θ*	3.63 (1.96, 6.63)	3.76 (2.04, 6.81)
**Data without 2016**		
*r*	0.052 km^−1^ (0.019, 0.065 km^−1^)	0.050 km^−1^ (0.038, 0.062 km^−1^)
*x*_50_/*x*_100_	− 45.08 km (− 66.89, − 31.32 km)	− 48.19 km (− 69.90, − 34.71 km)
*c*	10.39 km/year (6.41, 14.36 km/year)	10.37 km/year (6.40, 14.31 km/year)
*θ*	11.41 (6.92, 17.91)	11.64 (7.08, 18.27)

The values between the brackets are the lower and upper 95% confidence limits.

*CNE* constrained negative exponential.

Given the continuous expansion of the forefront of the infections and the significant increase of the resources devoted to the monitoring programs, the data from 2016 were substantially different from the other years’ data, with an uncharacteristically greater spatial extent of high levels of infection than in other years, including 2017 (Fig. 5). Therefore, we repeated the above analysis without the 2016 data. The estimated rates of movement of the disease front were changed slightly: 10.39 km per year (95% CI: 6.41–14.36 km per year) for the logistic and 10.37 km per year (95% CI: 6.40–14.31 km per year) for the CNE function (Table 3; Supplementary Analysis 1). Thus, including or excluding the 2016 data hardly influenced the estimated rates of movement of the disease front.

### Simulation of the Puglia sampling strategy

After simulating the sampling strategy with chosen input parameters and re-estimating these parameters from the simulated data, the mean bias (average difference between the estimated value and the model input value of the rate of movement of the front) and root-mean-squared error (RMSE) were calculated from the results (Table 4). The mean bias was lowest when fitting a logistic function for both the logistic function and CNE function in the input model. Mean bias was -0.039 km per year for the logistic input and 0.011 km per year for the CNE input, which are small values when compared to the actual accuracy of the estimates resulting from the analysis of empirical data. The RMSEs for these analyses were 0.53 km per year and 0.75 km per year respectively. This means that the logistic function is the best estimator for the rate of spread for both the logistic function and the CNE function in the input model.

**Table 4:** Mean bias and root-mean-squared error when estimating the rate of spread *c* in the Puglia sampling strategy simulation with Gallipoli as the assumed origin.

Function	Mean bias	RMSE
	(km/year)	(km/year)
**Input function: logistic**		
Logistic	− 0.039	0.53
CNE	− 0.15	0.77
**Input function: CNE**		
Logistic	0.011	0.75
CNE	− 0.13	0.79

Bias is the average difference between the estimated value of the rate of spread, *c*, and the true value of this rate in km/year.

*RMSE* root-mean-square error.

*CNE* constrained negative exponential.

### Invasion front properties

The above results indicate that the invasion front has the shape of a logistic curve which moves in north-western direction at a rate of 10.0 km per year (95% CL: 7.48–12.49 km per year). The width of the front is the distance between the 1st and 99th percentile, which is

9 $$x_{1} - x_{99} = \frac{{2{\text{log}}\left( {99} \right)}}{0.059} = 155.8 \;{\text{km}}$$

The distance between the 5th and 95th percentile was: $x_{5} - x_{95} = 99.8 {\text{km}}$. With knowledge about the width of the invasion front, the width of demarcated zones could be adapted, e.g. the width of the containment zone could be set to the width of the invasion front.

Using Eq. 6 to calculate $t$ for $x_{1}$ = 0 (i.e. the 1% percentile of the front is located at Gallipoli) we find for $r = 0.059$ km^−1^ $, x_{50} = - 25.76 {\text{km}},$ and $c = 10.0 {\text{km per year}}$ that $t \approx - 5.24$ years. In words: the 1% point of the front was located at Gallipoli in 2008 (5 years before 2013, the year of first detection). By repeating this calculation 1000 times with random numbers drawn from a normal distribution (with the estimated values for the means and standard deviations) for the parameters we calculated 95% CLs of −10.27 years and −0.21 years. This means that according to our findings, it is possible that the spread of OQDS through Puglia did not start in 2013, but approximately 5 years earlier, but with a wide margin of error of plus or minus 5 years.

### Sensitivity analysis for the point of origin of the epidemic

In the sensitivity analysis of the point of origin, we found that the estimated rate of spread increases to 17.33 km per year (95% CI: 14.80–19.95 km per year) when Santa Maria di Leuca is used as point of origin, 15.74 km per year (95% CI: 13.29–18.40 km per year) when Otranto is used as origin, and 14.21 km per year (95% CI: 12.11–16.41 km per year) when Maglie is used as origin (Table 5; Supplementary Analysis 2).

**Table 5:** Rate of spread estimates of the logistic and constrained negative exponential functions when assuming different points of origin in the analysis of the Puglia data (see Supplementary Analysis 2).

Analysis point of origin	Function	
	Logistic (Rate of spread in km/year)	CNE (Rate of spread in km/year)
Gallipoli	9.95 (7.48, 12.49)	10.19 (7.74, 12.63)
Santa Maria di Leuca	17.33 (14.80, 19.95)	17.58 (14.28, 20.00)
Otranto	15.74 (13.29, 18.40)	16.26 (13.47, 19.22)
Maglie	14.21 (12.11, 16.41)	14.35 (12.27, 16.55)

The values between the brackets are the lower and upper 95% confidence limits in km/year.

*CNE* constrained negative exponential.

After generating data with the alternative points of origin and analysing the data assuming Gallipoli as the point of origin, we calculated the mean bias and RMSE of this analysis (Table 6; Supplementary Analysis 3). The results show that these analyses give lower estimates of the rate of movement of the front. The underestimation is similar in magnitude to the overestimations when using these towns as the origin of the invasion in the data analysis of our actual data set (Supplementary Analysis 2). Additionally, we find high RMSEs. These analyses indicate that the choice for point of origin has a large impact on the parameter estimations.

**Table 6:** Mean bias and root-mean-square error of the Puglia sampling strategy simulation with a logistic function for different points of origin of the simulated disease spread and fitted with Gallipoli as assumed origin (see Supplementary Analysis 3).

Simulation point of origin	Mean bias (km/year)	RMSE (km/year)
Gallipoli	− 0.039	0.53
Santa Maria di Leuca	− 6.73	7.76
Otranto	− 4.16	7.16
Maglie	− 2.36	2.64

*RMSE* root-mean-squared error.

Bias is the average difference between the estimated value and the true value of the rate of movement of the front in km/year.

## Discussion

We analysed the spread of *X. fastidiosa* in Puglia based on sampling for *X. fastidiosa* presence in the region performed between 2013 and 2018. The shape of the invasion front of the *X. fastidiosa* invasion in Puglia was most accurately described with a declining logistic function. The estimated rate of movement of this front (i.e. the rate of spread of the invasion) was 10.0 km per year (95% CI: 7.5–12.5 km per year) (Table 3 & Fig. 6). To our knowledge this is the first analysis attempting to find the best fitting shape of an invasion front based on empirical data. This is also the first empirical estimate of the rate of disease spread of the *X. fastidiosa* invasion in Puglia. Our findings also show that the invasion of *X. fastidiosa* in Puglia possibly did not start in 2013, but approximately five years earlier. This is consistent with previous reports that the spread of *X. fastidiosa* in a region has already been ongoing for multiple years before it was discovered^12,31^.

Comparing our findings with the rate of spread estimate of Abboud et al.^14^, of 1.9 km per year highlights a characteristic difference in disease spread of a pathogen in different environments. Knowledge on disease spread in one environment cannot easily be extrapolated to provide knowledge on disease spread in a characteristically different environment.

Knowing the width of the invasion front could change the way we should look at the demarcated infected and containment zones. The infected zone is assumed to have a high proportion of infected trees and the containment zone is assumed to have this proportion gradually declining from its border with the infected zone (a high proportion of infected trees) to its border with the buffer zone (a proportion of zero). However, if the width of the invasion front is approximately 156 km, this would indicate that the width of a containment zone should also have approximately this width. Additionally, sampling in the infected zone was stopped from 2016 onwards, because this area was assumed to be lost to the disease. Yet, our findings also show that the proportion of infected trees in the infected zone is not as high as is assumed, as the proportion of positive values drops already close to Gallipoli (Fig. 6, where Gallipoli is at Distance = 0 km).

Although the rate of spread of the invation might be estimated accurately enough with this analysis method, the fit of the models does not look optimal. Comparing Fig. 6 with Fig. 5a, the 2016, 2017, and 2018, lines are quite far from their optimal fit by being forced in a sequence with a set distance from the other years. Especially the data for 2017 seems to be under-estimated when a single disease front progress curve is fitted to the data over time. This discrepancy may be a result of the different sampling designs adopted throughout time. For example, in accordance with the promulgation of the Commission Implementing Decision (EU) 2015/789 surveillance was intensified after 2015 and based on the demarcation of the whole affected territory, in contrast with the previous monitoring programs targeting single foci, generating scattered data. From 2016 onwards, sampling was more structured in number and sample choice, with sampling done almost exclusively in the buffer and containment zones, whose borders changed three times since 2016. If the shape of the dispersal front and the rate of movement of the front of *X. fastidiosa* in Puglia stay the same over time^28,29^, forcing the model to have the same shape in all years and shifting over the x-axis with the same amount every year, might wrinkle out a lot of inconsistencies from the sampling. Although there might be large differences between years in the dataset when models are fitted with different shapes and locations (Fig. 5), making it hard to interpret the invasion pattern, the average estimated shape of the invasion front and the rate of movement could very well be a good approximation of the actual shape and rate of movement of the front. Additionally, we have found that including or excluding 2016 data (which is inconsistent compared to other years) does not have a large impact on the results (Supplementary Analysis 1). The stochastic simulations confirmed that the shape and rate of the front can be retrieved from data collected using the Puglia sampling strategy (Table 5). This shows that the inconsistent sampling might not cause a large problem. However, there is also a sampling bias with respect to which trees are chosen to be sampled in a location. Trees that show possible disease symptoms are preferentially chosen to be sampled, and at a location where multiple trees show possible symptoms, more samples may be taken. This could mean that there is a bias for more positive samples (infected trees). Furthermore, over the years there have been varying control strategies applied in different parts of Puglia^3,19,32–34^. This spatio-temporal change could have affected the disease spread and therefore our parameter estimations. Future surveillance strategies might take into account possible future use of the data for estimating rates of spread. Best estimates are obtained when random sampling is applied. Random sampling is not necessarily contradictory to the needs of surveillance. It might be sufficient to “tag” each sample as “random”, or “non-random”, while in the latter case, a reason for sampling the tree might be indicated. This would provide useful information for analysis.

Because there is no certainty about Gallipoli being the origin of OQDS spread, we analysed alternative points of origin in a sensitivity analysis. We (1) analysed the real data from different points of origin, and (2) simulated new data using different points of origin and analysing this data assuming the epidemic started from Gallipoli. The results from both approaches indicate that choosing a different point of origin can significantly change the estimated parameters (Supplementary Analysis 2 and Supplementary Analysis 3). The alternative locations are further away from the assumed direction of the disease spread (North-West, land inwards), which could explain the increase in rate of spread, as the increase in distance needs to be compensated. This would also explain why choosing Santa Maria di Leuca as the disease origin has the highest estimated rate of spread. Additionally, we found high RMSEs in the simulation with alternative origins, which is to be expected, since there is a large discrepancy between the assumed point of origin and the true point of origin, making the data harder to fit. Together, these results indicate that choosing the correct point of origin is very important to get the right results. There is also reason to presume that the model resulting in the lowest rate of spread has identified the true origin of spread. In our case, estimates are lowest from Gallipoli, giving credibility to the assertion that Gallipoli is the true origin of the epidemic. In the Gallipoli area, one of the main Italian hubs for the commercialization of ornamental plants is located, supporting that it is a plausible location for entry of a new plant pathogen.

Because of the inconsistent sampling described above, analysing the data is no trivial task. For instance, an analysis of original point data did not yield any tangible results, presumably because the signal in the data was overshadowed by the variability and the irregularities in the sampling. Therefore, we aggregated the data in concentric distance circles around the origin to calculate the proportion of diseased trees in different distance classes so as to aggregate the information and filter out noise and amplify the signal. We showed here that this is an effective method to deal with inconsistent data that produces in our case small bias and RMSE in stochastic simulations of the sampling process. To fill up gaps in the data, we tried multiple methods of information transfer between years (e.g. including positive measurements from one year in all subsequent years, or including negative measurements from a year in all prior years), as well as trying different temporal cut-off points between years (instead of January 1^st^, which we used now, we also attempted cutting of between monitoring seasons at a cut-off date of April 1^st^ in each year). The stochastic simulations showed that these methods increased the mean bias of the analysis. The analysis as described in this paper had the smallest mean bias and thus gave the most accurate estimation of the rate of disease spread of all the methods tried.

Expert knowledge elicitation (EKE) by the European food safety authority (EFSA) resulted in the statement that 90% of the newly infected trees within a year lie within 5.2 km of a previously infected area (with a 95% CI of 0.7–14.0 km)^19^. The rate of movement of the disease front we found is about twice as large as the median estimate of 5.2 km per year of the EKE (although it does fall within the 95% CI range of the EKE estimation). However, there is a difference in definition between the rate of spread we estimated, and the distance an infection spreads within a year as estimated by EFSA. The number we estimated is the radial rate of range expansion, i.e. the rate of movement of the invasion front^18,25^. The parameter estimated by EFSA reflects disease dispersal rather than movement of the disease front. Furthermore, the estimate made by EFSA considered spread outside Puglia in the future, while our retrospective analysis addressed the observed spread in the past within Puglia. Thus, the movement rate found in our analysis and the spread rate assessed by EFSA^19^ do not measure the exact same ecological phenomenon and are therefore only approximately comparable. However, the two estimates and their uncertainty ranges indicate that they are of similar order of magnitude.

*X. fastidiosa* keeps spreading through Europe, as multiple countries are facing localized outbreaks or endemic or epidemic spread of the bacterium. However, recent molecular investigations clearly showed that no genetic correlations exist among isolates recovered in these outbreaks. More likely, they are the result of multiple and independent introductions from Central America^35^.

Currently, more data regarding the invasion of this bacterium in Puglia is being gathered, as the buffer zone is continuously sampled for the presence of this bacterium. In taking these samples, we advocate that any sample taken should be labelled as “random” or “non-random” to distinguish those trees that were randomly selected from the population of trees at a location, and trees that were selected specifically because they did, or did not, show symptoms. The methods outlined in this and other papers can be used on existing and future data to re-estimate the parameters of the models^12,14^. In this way, a more accurate estimate could be made, or a change in the pattern could be detected. The results of the parameter estimates can be used in models that predict the future spread of *X. fastidiosa* in the region, and possibly be adapted to forecast the spread in other areas. The parameters and such models can be used to aid management decisions on containing and preventing the spread of *X. fastidiosa* and possibly other plant diseases.

## Supplementary Information

Supplementary Information 1.

Supplementary Information 2.

Supplementary Information 3.

## Data Availability

The complete dataset is available online at https://github.com/DBKottelenberg/OQDS_Xf_Puglia.

## Code availability

The details on the data analysis are available in the R script online at https://github.com/DBKottelenberg/OQDS_Xf_Puglia.
