# Supplementary material for: Shape and rate of movement of the invasion front of Xylella fastidiosa spp. pauca in Puglia
Source: Sci Rep. 2021 Jan 13;11:1061. doi: 10.1038/s41598-020-79279-x (PMC7806996; doi:10.1038/s41598-020-79279-x)
Supplement: Supplementary file 1 — Supplementary Information 1. [file 41598_2020_79279_MOESM1_ESM.pdf]

Shape and rate of spread of the invasion front of *Xylella fastidiosa* spp. *pauca* in Puglia

Supplementary Analysis 1: Analysing dataset without 2016

David Kottelenberg Lia Hemerik Maria Saponari Wopke van der Werf

In this analysis we will repeat the analysis where we estimate the shape of the front and the rate of spread, except we leave out data from 2016 since this data is characteristically different from the other years. The code used for the analysis is the same as the code found in *Shape of the Front.R* and *Rate of spread.R* on Github (https://github.com/DBKottelenberg/OQDS_Xf_Puglia) except that data for the year 2016 has been left out.

Shape of the Front

In the first part, we estimate the best fitting shape for the invasion front. We do this by fitting a deterministic model (negative exponential function, a logistic function, or a constrained negative exponential function (see main text, materials and methods)) with a stochastic model (binomial distribution or beta-binomial distribution) to the data. We optimize the parameters for these models for every year separately. We then calculate the AIC of every fitted combination of deterministic and stochastic models, sum the AICs of all the years, and compare the AICs between every model. The lowest AIC is the best fitting shape of the front.

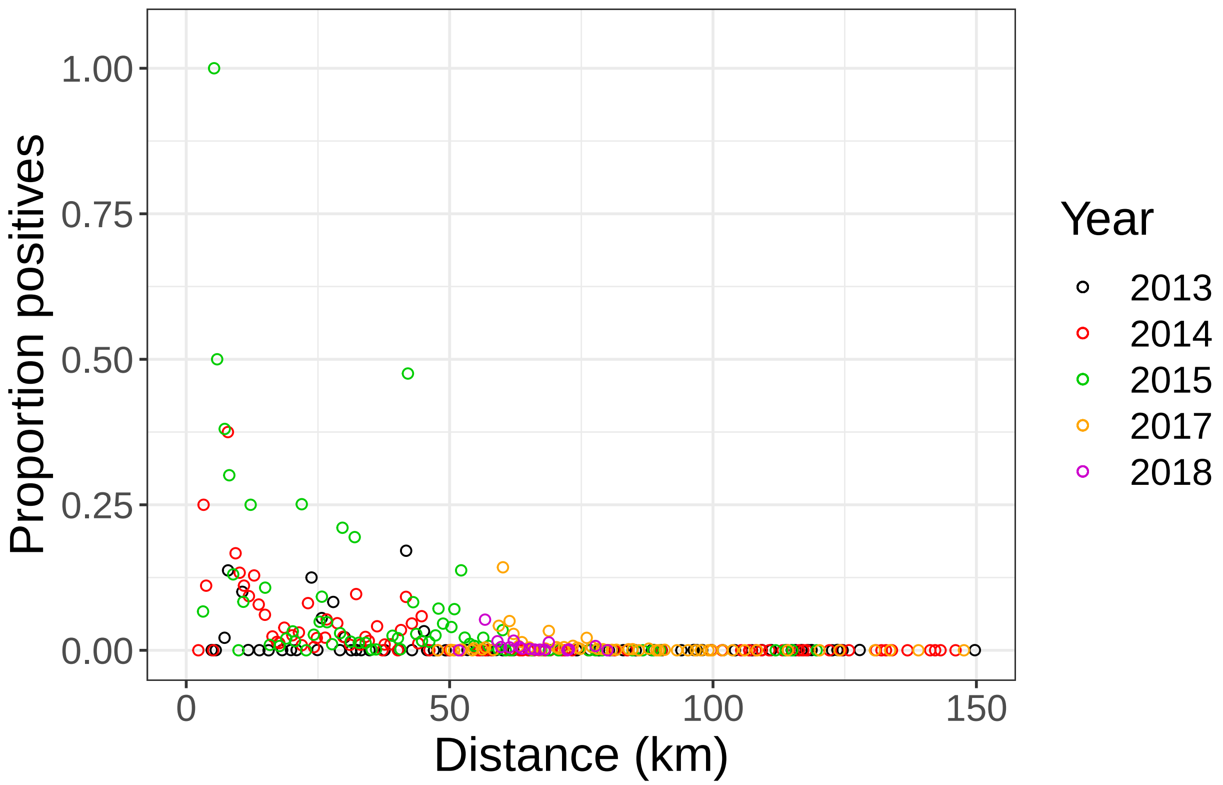

Figure S1. The data without 2016. X−axis: distance in km (distance circle). Y−axis: Proportion of positive samples.

Deterministic: Negative exponential; Stochastic: Binomial distribution

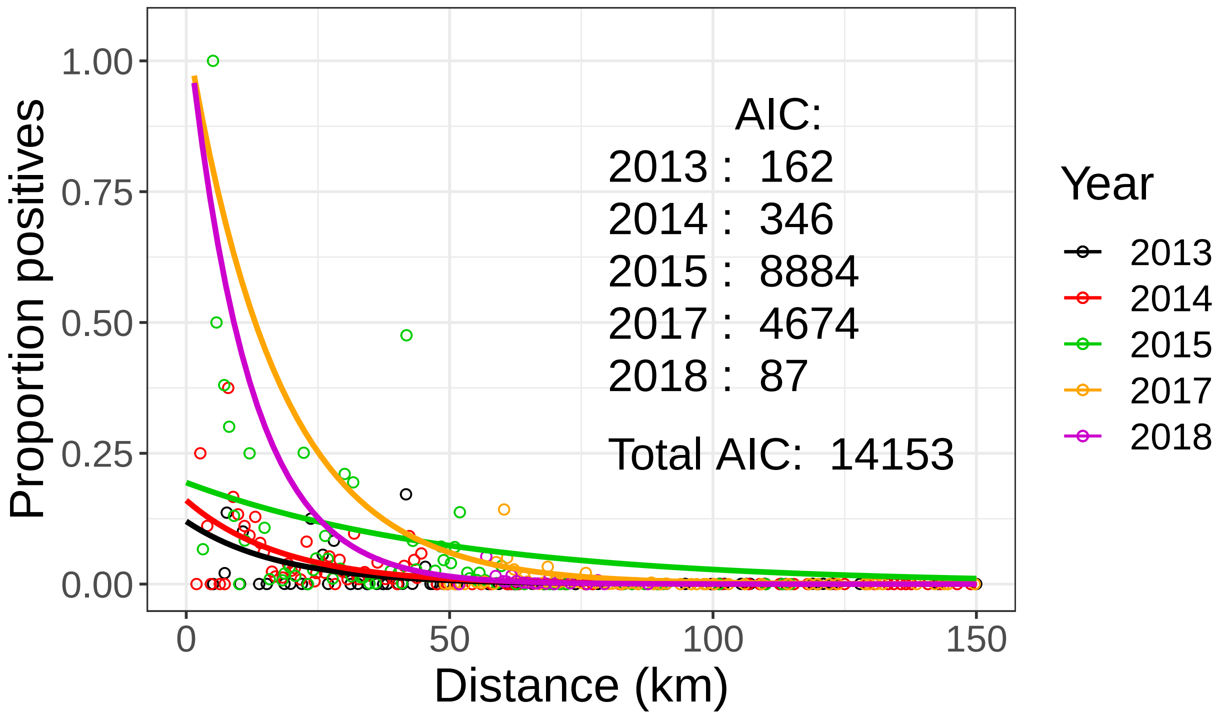

Figure S2. Negative exponential functions fitted on the data with a binomial stochastic distribution. X−axis: distance in km (distance circle). Y−axis: Proportion of positive samples.

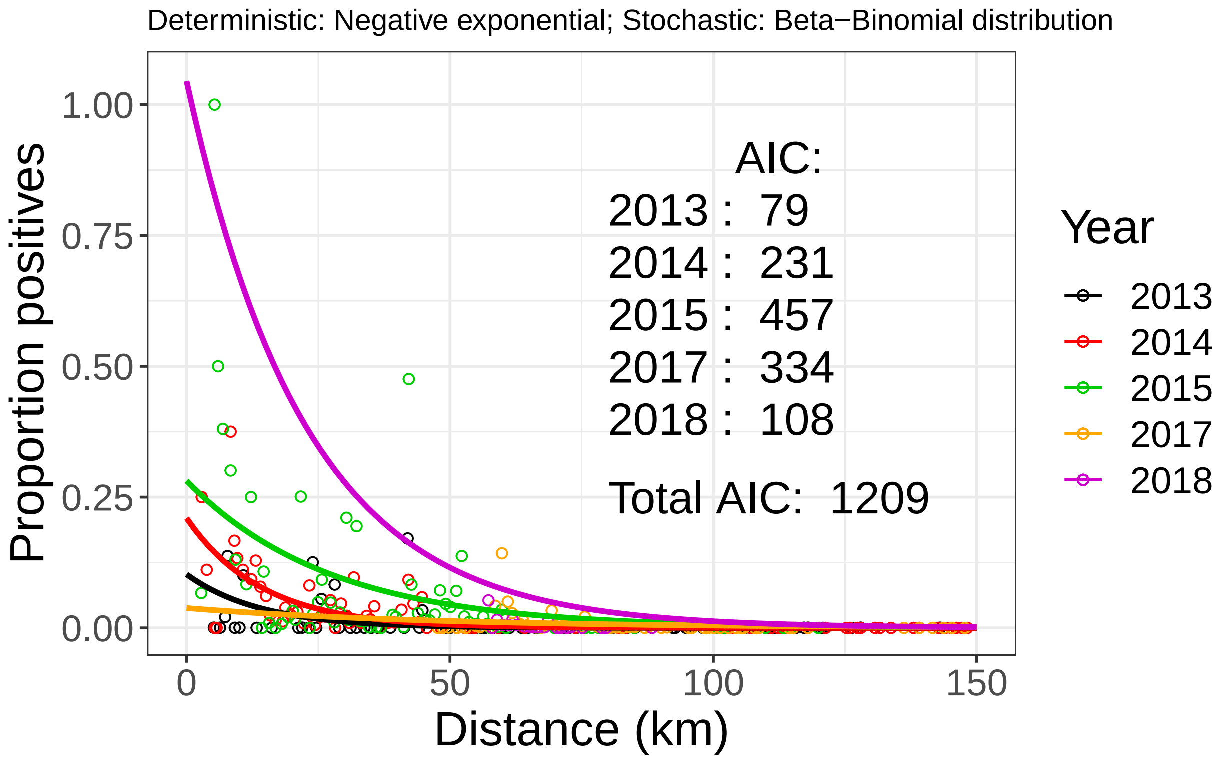

Figure S3. Negative exponential functions fitted on the data with a beta−binomial stochastic distribution. X−axis: distance in km (distance circle). Y−axis: Proportion of positive samples.

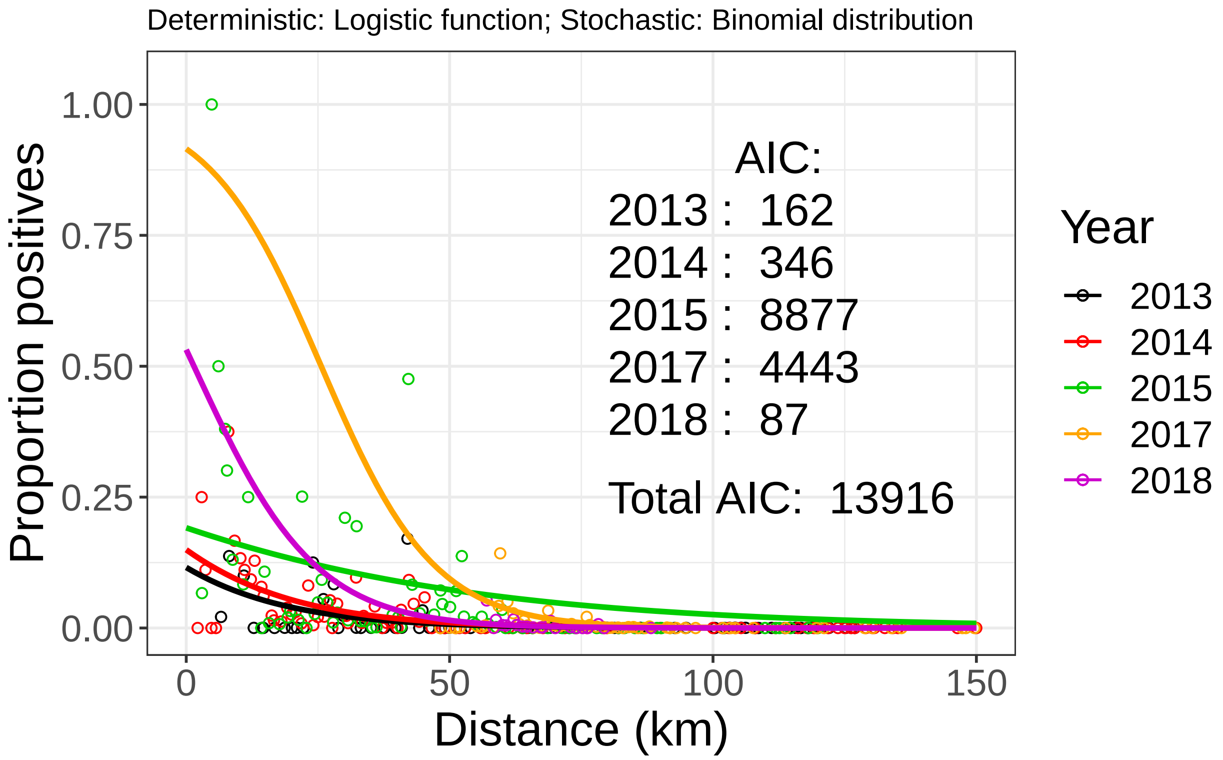

Figure S4. Logistic functions fitted on the data with a binomial stochastic distribution. X−axis: distance in km (distance circle). Y−axis: Proportion of positive samples.

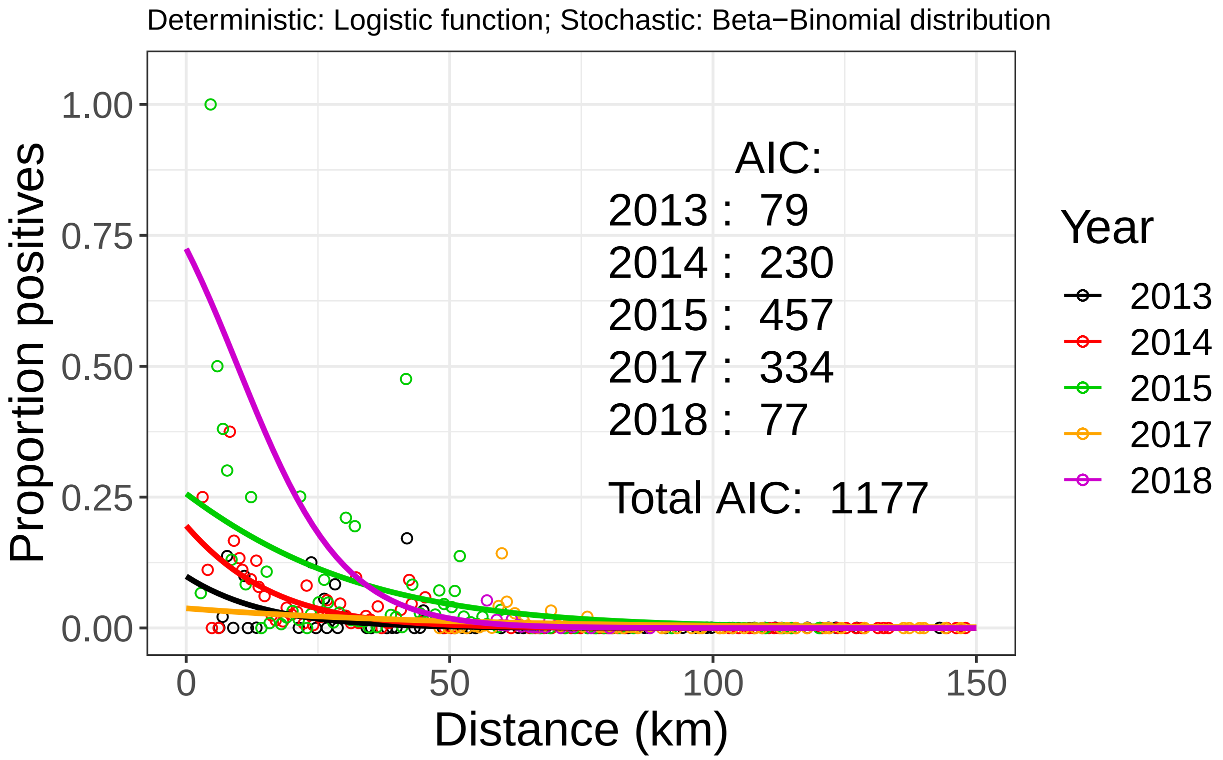

Figure S5. Logistic functions fitted on the data with a beta−binomial stochastic distribution. X−axis: distance in km (distance circle). Y−axis: Proportion of positive samples.

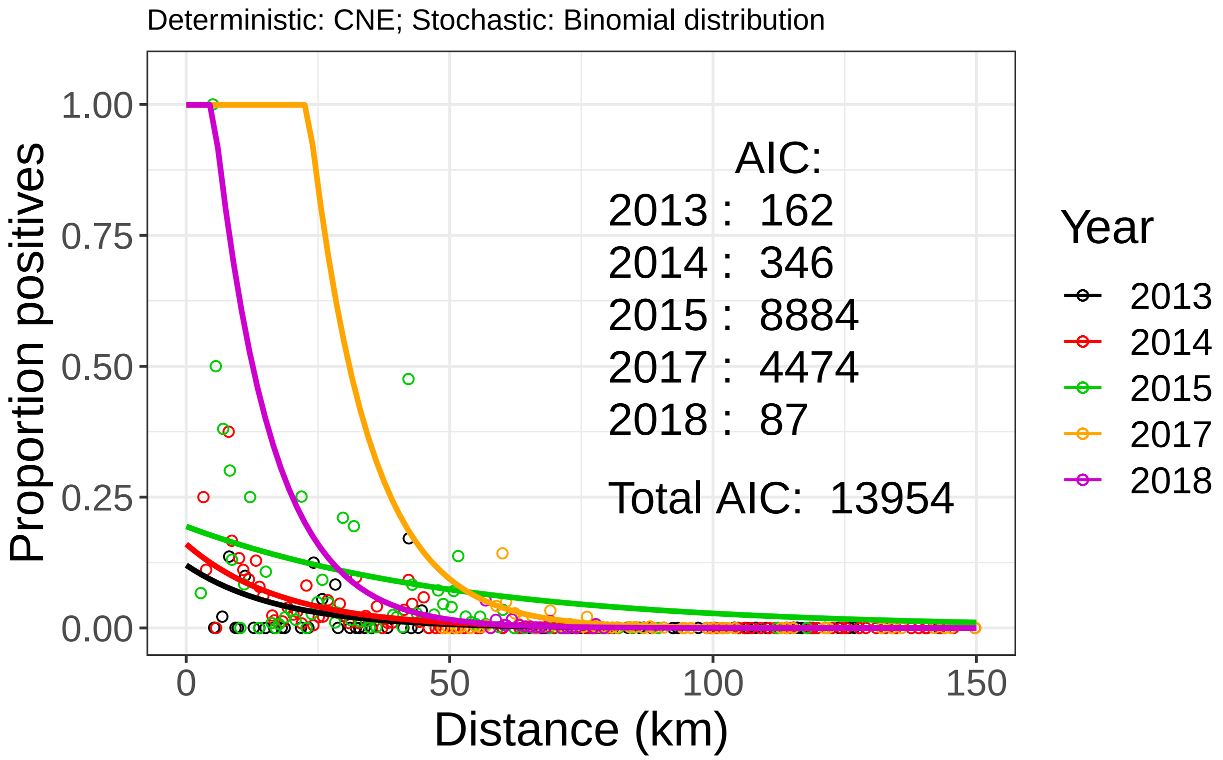

Figure S6. CNE functions fitted on the data with a binomial stochastic distribution. X−axis: distance in km (distance circle). Y−axis: Proportion of positive samples.

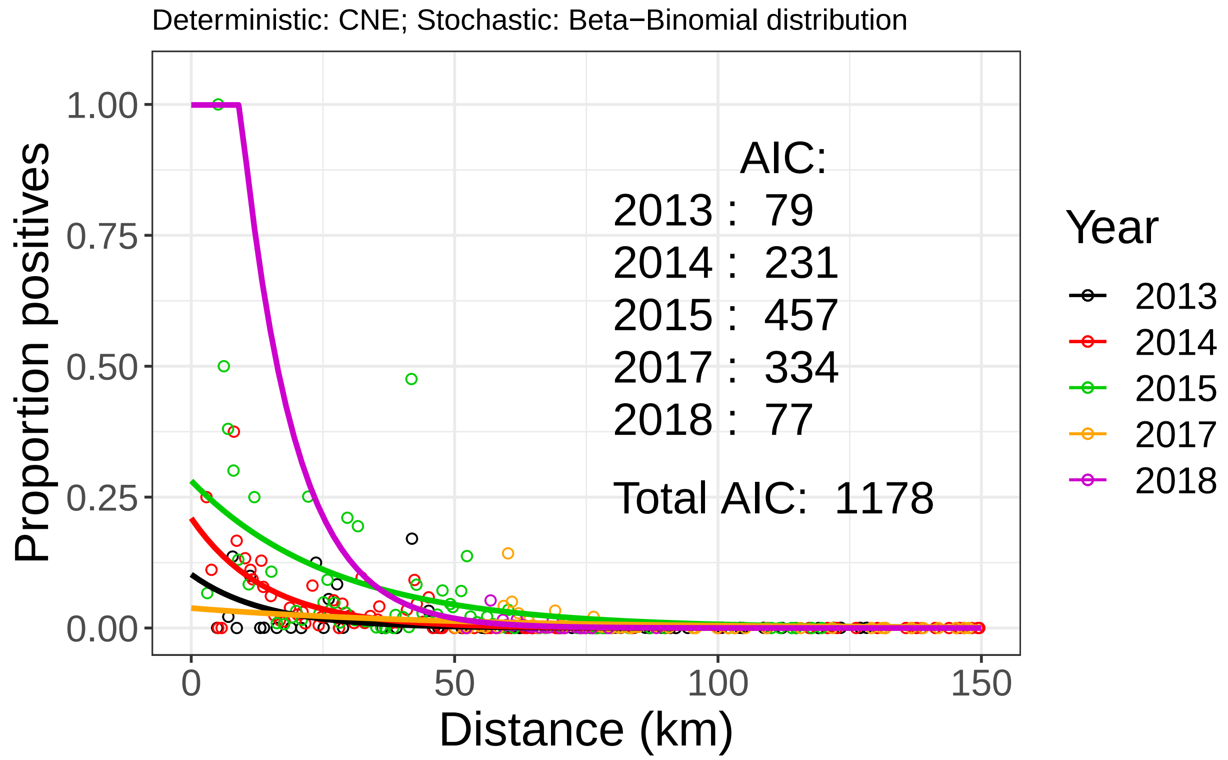

Figure S7. CNE functions fitted on the data with a beta−binomial stochastic distribution. X−axis: distance in km (distance circle). Y−axis: Proportion of positive samples.

Rate of Spread

In the second part, we estimate the rate of spread of the invasion front by assuming a fixed distance between the fitted models for every year. This way, one set of parameters can be estimated for 2013 - 2018. Because the logistic and CNE functions combined with the beta-binomial distribution are the best fitting shapes, we estimate the parameters for these models only.

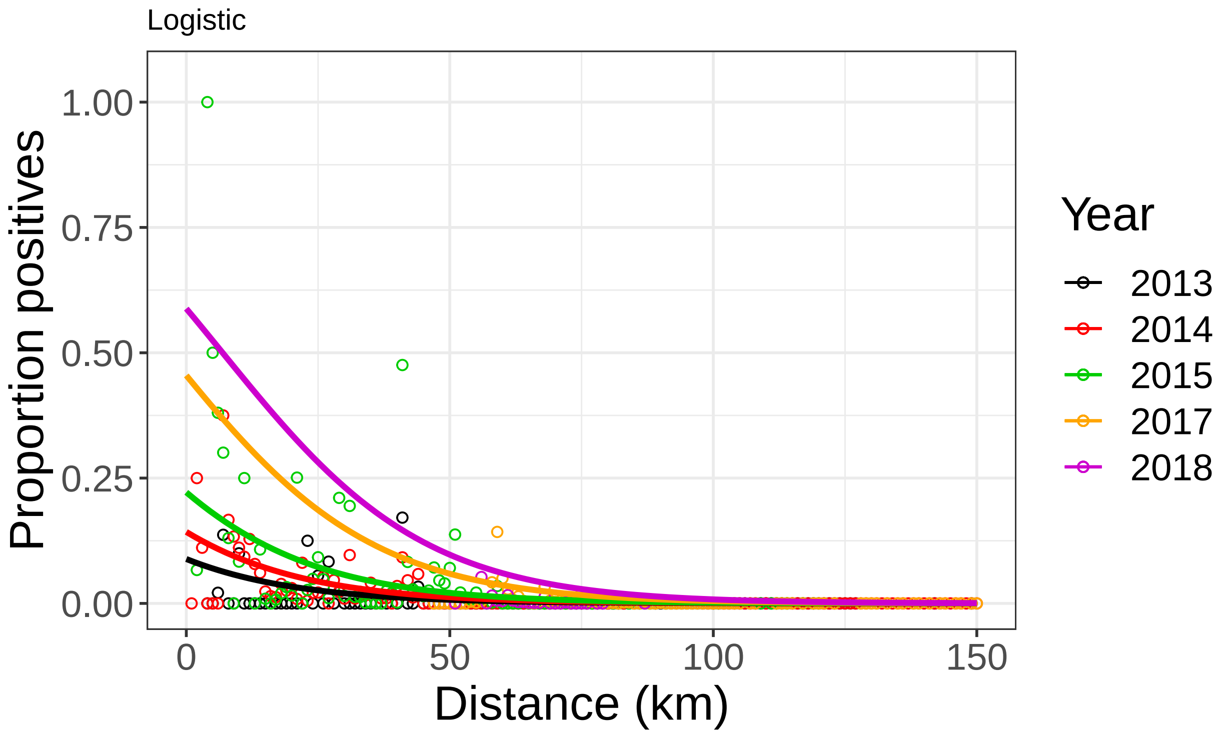

Figure S8. Logistic functions fitted on the data with
a beta−binomial stochastic distribution. The functions for every year are fit in
sequence with a fixed distance between years . X−axis: distance in km (distance circle).
Y−axis: Proportion of positive samples.

```
## [1] "Parameter estimations and their lower and upper 95% confidence limits (CLs)"

##            r lower 95% CL upper 95% CL
##   0.05171461   0.03934349   0.06468875

##          x50 lower 95% CL upper 95% CL
##    -45.07788    -66.89000    -31.31648

##            c lower 95% CL upper 95% CL
##     10.39263      6.40984     14.36017

##        theta lower 95% CL upper 95% CL
##    11.405048     6.923725    17.911928
```

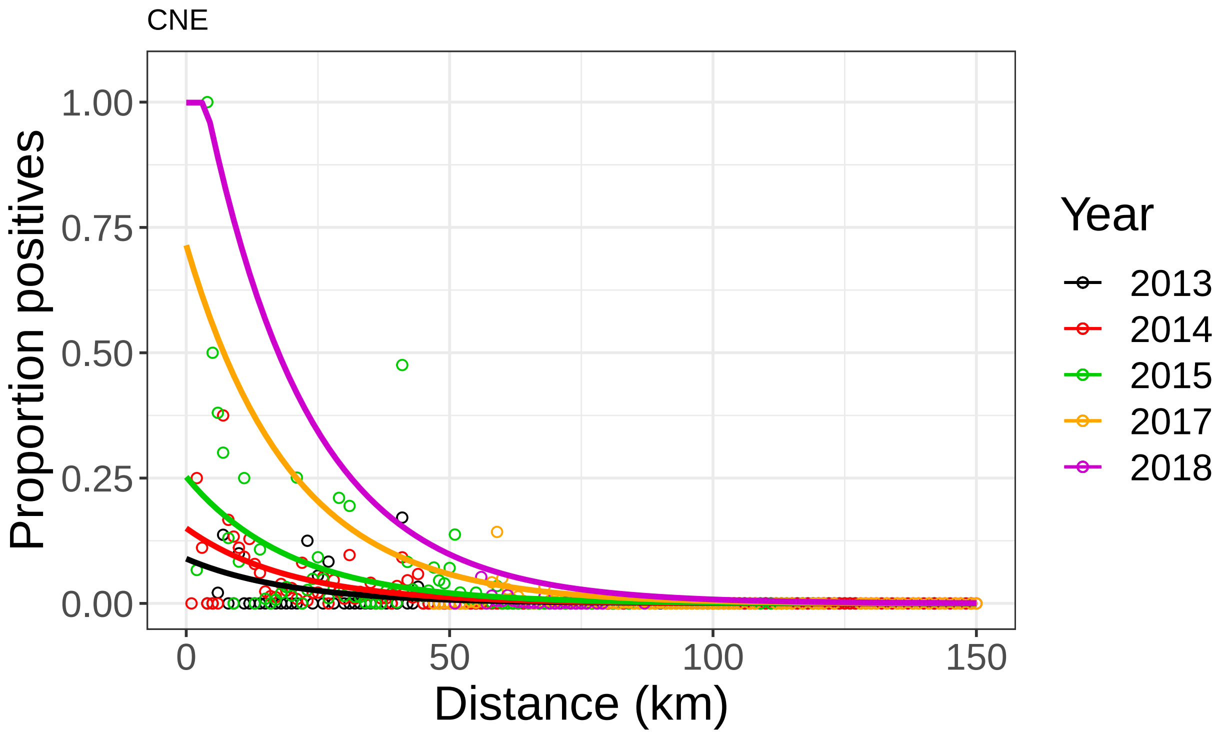

Figure S9. CNE functions fitted on the data with a beta−binomial stochastic distribution. The functions for every year are fit in sequence with a fixed distance between years . X−axis: distance in km (distance circle). Y−axis: Proportion of positive samples.

```
## [1] "Parameter estimations and their lower and upper 95% confidence limits (CLs)"

##              r lower 95% CL upper 95% CL
##     0.05018257   0.03844386   0.06240261

##           x100 lower 95% CL upper 95% CL
##      -48.18594    -69.90260    -34.71048

##              c lower 95% CL upper 95% CL
##      10.371925     6.403151    14.309216

##          theta lower 95% CL upper 95% CL
##      11.641821     7.083475    18.270302
```

Summary of Results

For convenience, the above results are summarized in Tables S1 - S2.

Comparing these results with the results that do include 2016, we see that the shape of the front is still a logistic or CNE curve. We also see that the rate of spread did increase slightly, but not significantly. The only large change we see is the increase in the theta parameter. This increase can be explained by the fact that the 2016 data was characteristically different from the other data, having a lot of points at a proportion of 1.0. Removing this data decreases the overdispersion, which is reflected in the increase of the theta parameter.

Table S1. The AIC's for models fitted without 2016 data.

Year	Binomial			Beta-binomial		
	Negative exponential	Logistic	CNE	Negative exponential	Logistic	CNE
2013	162	162	162	**79**	**79**	**79**
2014	346	346	346	231	**230**	231
2015	8884	8877	8884	**457**	**457**	**457**
2017	4674	4443	4474	**334**	**334**	**334**
2018	87	87	87	108	**77**	**77**
Total	14153	13916	13954	1209	**1177**	1178

Table S2. The results of the parameter estimations without 2016 data. The values between the brackets are the 95% confidence limits (CL's)

Parameter	Logistic	CNE
r	0.052 (0.019, 0.065)	0.050 (0.038, 0.062)
x_{50}/x_{100}	-45.08 (-66.89, -31.32)	-48.19 (-69.90, -34.71)
c	10.39 (6.41, 14.36)	10.37 (6.40, 14.31)
theta	11.41 (6.92, 17.91)	11.64 (7.08, 18.27)
