# Supplementary material for: Shape and rate of movement of the invasion front of Xylella fastidiosa spp. pauca in Puglia
Source: Sci Rep. 2021 Jan 13;11:1061. doi: 10.1038/s41598-020-79279-x (PMC7806996; doi:10.1038/s41598-020-79279-x)
Supplement: Supplementary file 2 — Supplementary Information 2. [file 41598_2020_79279_MOESM2_ESM.pdf]

Shape and rate of spread of the invasion front of *Xylella fastidiosa* spp. *pauca* in Puglia

Supplementary Analysis 2: Analysis of the data with alternative assumed disease origins

David Kottelenberg Lia Hemerik Maria Saponari Wopke van der Werf

Analysis of the shape of the front and the rate of spread from other points of origin

In this analysis estimate of the shape of the invasion front and the rate of spread of this front for assumed origins other than Gallipoli. This gives an indication of the importance of choosing the correct point of origin. We have chosen the following alternative origins:

- Santa Maria di Leuca (longitude: 18.359826; latitude: 39.796772)
- Otranto (longitude: 18.490599; latitude: 40.146590)
- Maglie (longitude: 18.299162; latitude: 40.121722)

The code used for the analyses of these origins is the same as the code found in *Rate of spread.R* on Github (https://github.com/DBKottelenberg/OQDS_Xf_Puglia) except for the coordinates used, which is the coordinates as above for every origin. The methods of these analyses are the same as explained in the main text (materials and methods) and Supplementary Analysis 1, except for the difference in assumed point of origin and the inclusion of 2016.

For each assumed place of origin different from Gallipoli we present the fit of: 1 - a negative exponential function with binomial error to data of separate years.
2 - a negative exponential function with beta-binomial error to data of separate years.
3 - a logistic function with binomial error to data of separate years.
4 - a logistic function with beta-binomial error to data of separate years.
5 - a constrained negative exponential function (CNE) with binomial error to data of separate years.
6 - a CNE with beta-binomial error to data of separate years.
7 - a logistic function with beta-binomial error to data of all years together assuming a fixed distance between the yearly functions.
8 - a constrained negative exponential function (CNE) with binomial error to data of all years together assuming a fixed distance between the yearly functions.
For the formulas of the exponential, logistic, and constrained negative exponential we refer to Table 1 in the main text. For the description of the parameters we refer to lines 231-237 and 247-259 in the main text.

1. Santa Maria di Leuca

Shape of the Front

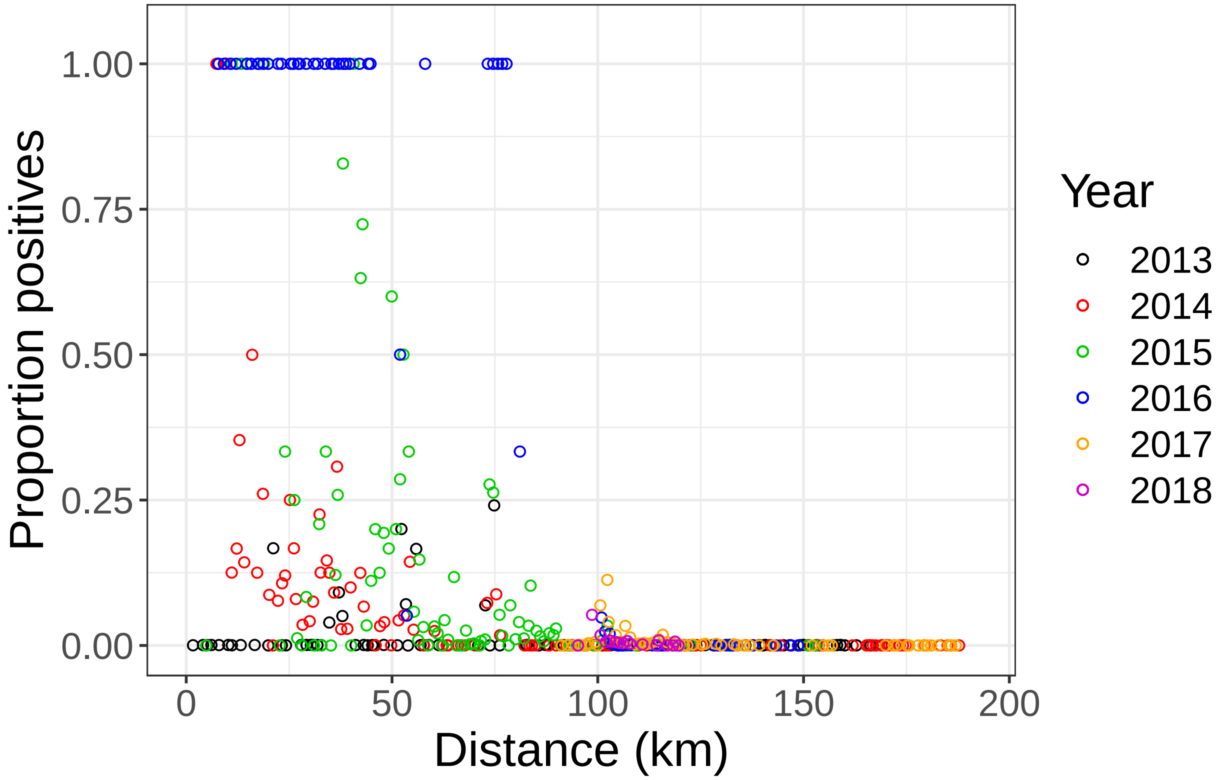

Figure S10. The data when the point of origin is assumed to be Santa Maria di Leuca. X−axis: distance in km (distance circle). Y−axis: Proportion of positive samples.

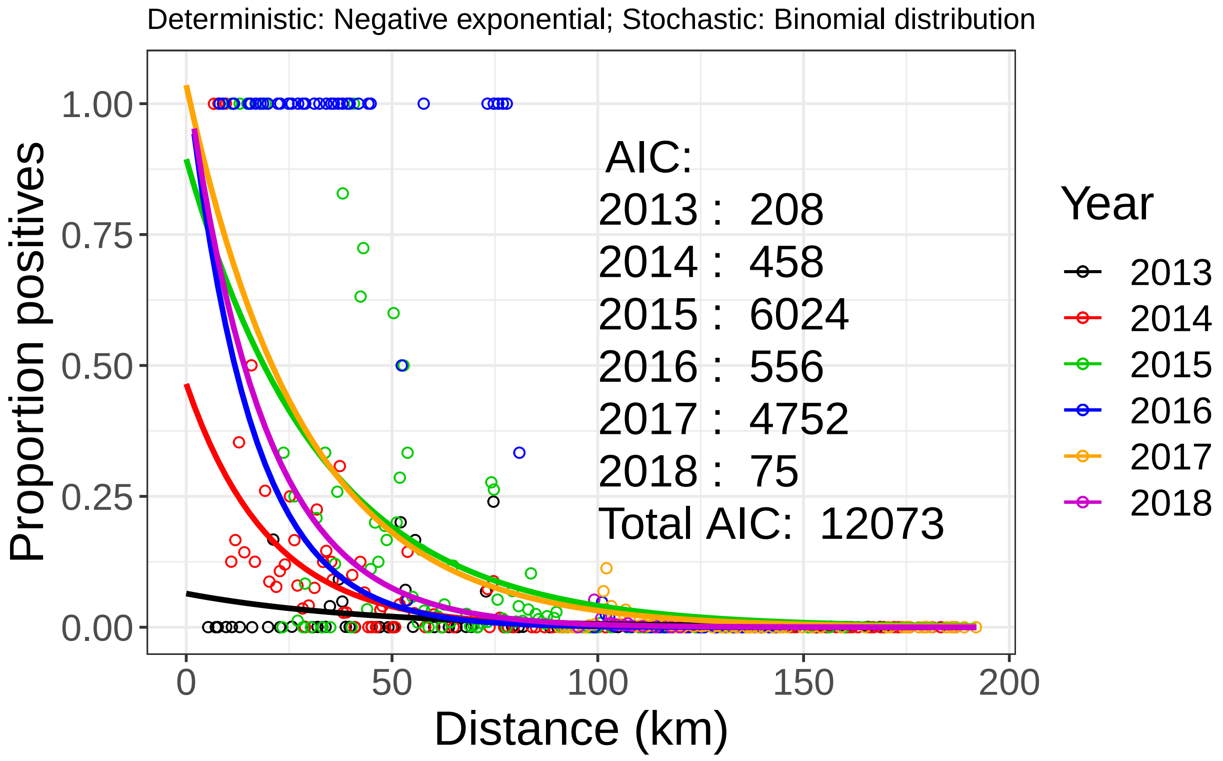

Figure S11. Negative exponential functions fitted on the data with a binomial stochastic distribution. The point of origin is assumed to be Santa Maria di Leuca. X−axis: distance in km (distance circle). Y−axis: Proportion of positive samples.

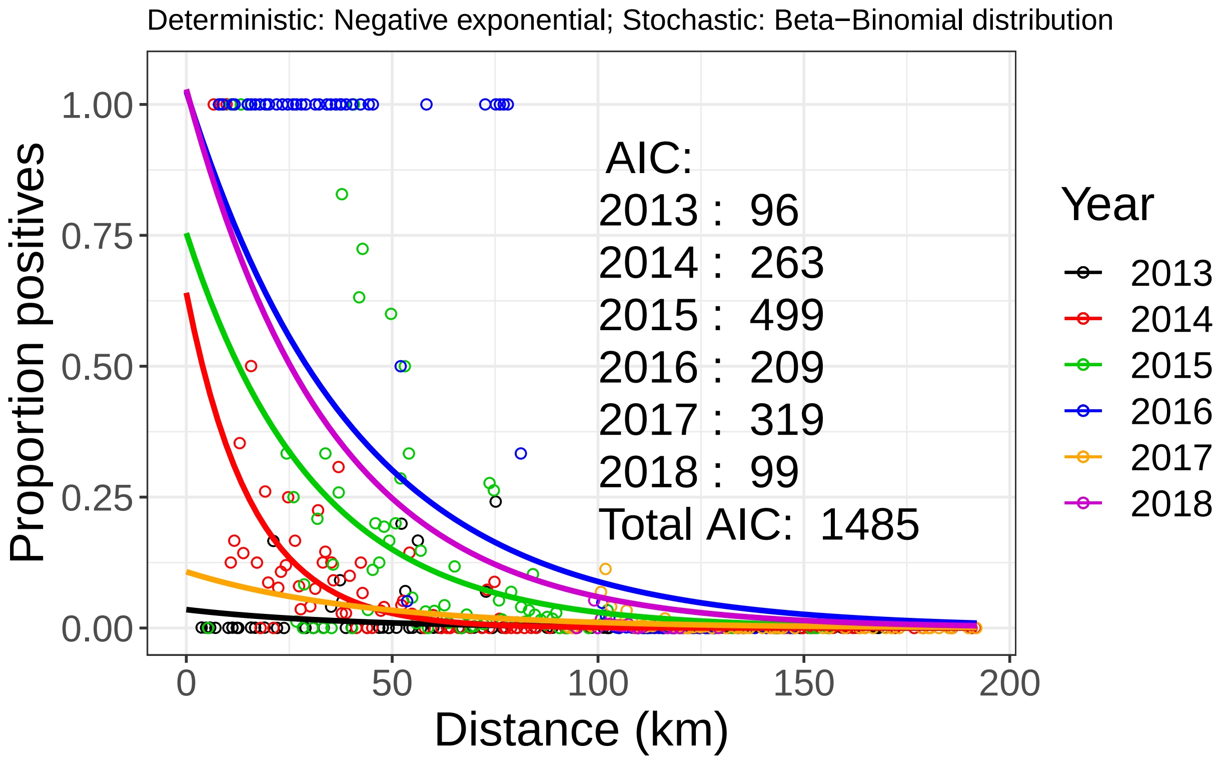

Figure S12. Negative exponential functions fitted on the data with a beta−binomial stochastic distribution. The point of origin is assumed to be Santa Maria di Leuca. X−axis: distance in km (distance circle). Y−axis: Proportion of positive samples.

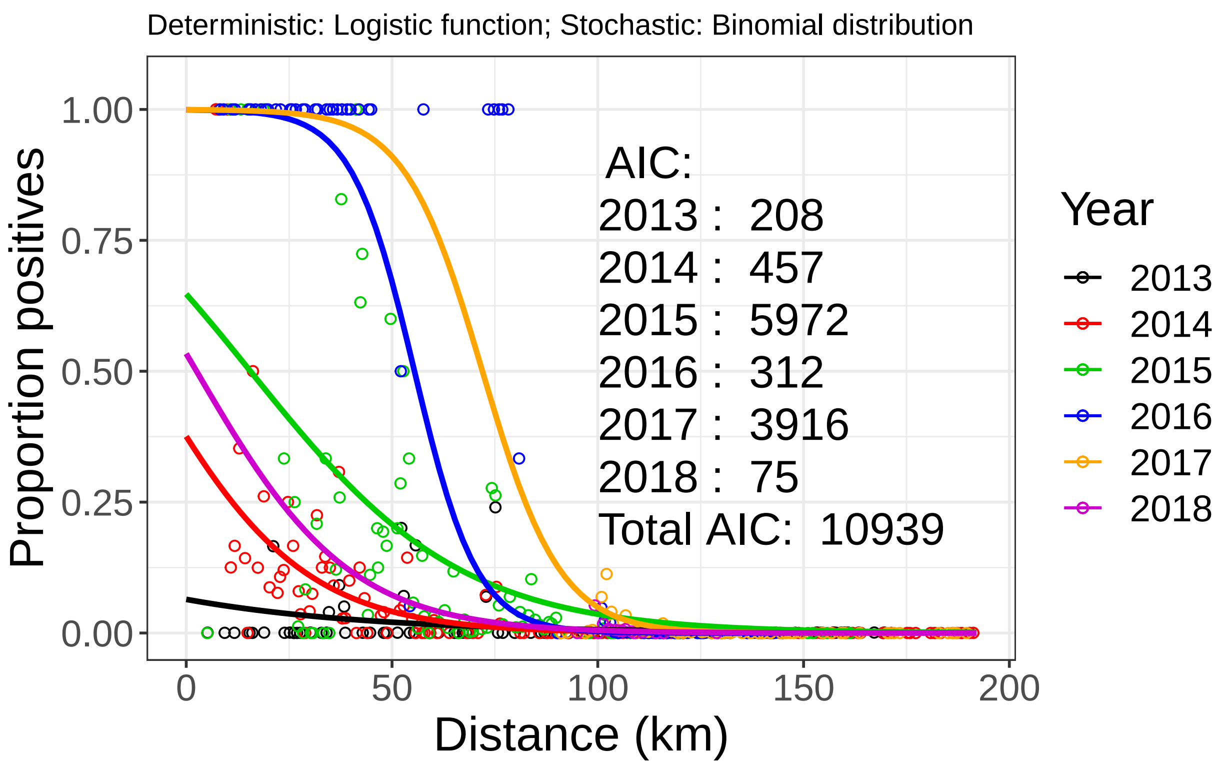

Figure S13. Logistic functions fitted on the data with a binomial stochastic distribution. The point of origin is assumed to be Santa Maria di Leuca. X−axis: distance in km (distance circle). Y−axis: Proportion of positive samples.

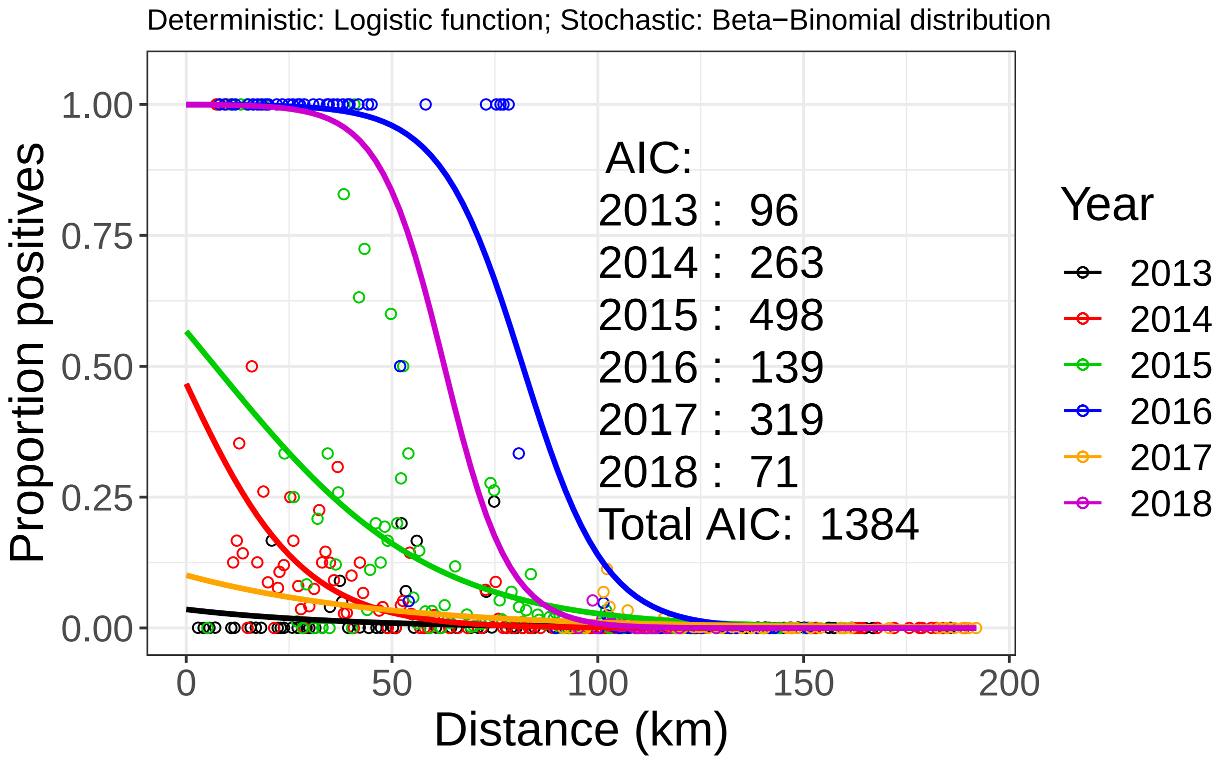

Figure S14. Logistic functions fitted on the data with a beta−binomial stochastic distribution. The point of origin is assumed to be Santa Maria di Leuca. X−axis: distance in km (distance circle). Y−axis: Proportion of positive samples.

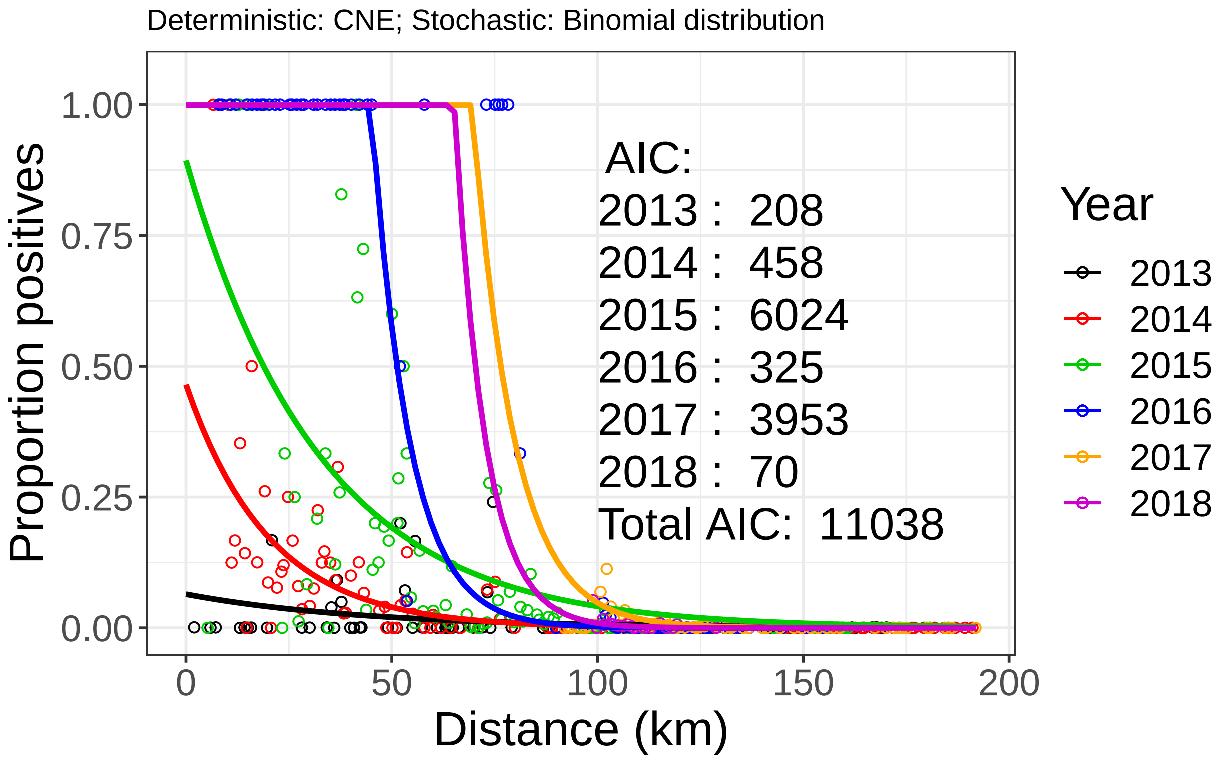

Figure S15. CNE functions fitted on the data with a binomial stochastic distribution. The point of origin is assumed to be Santa Maria di Leuca. X−axis: distance in km (distance circle). Y−axis: Proportion of positive samples.

Deterministic: CNE; Stochastic: Beta−Binomial distribution

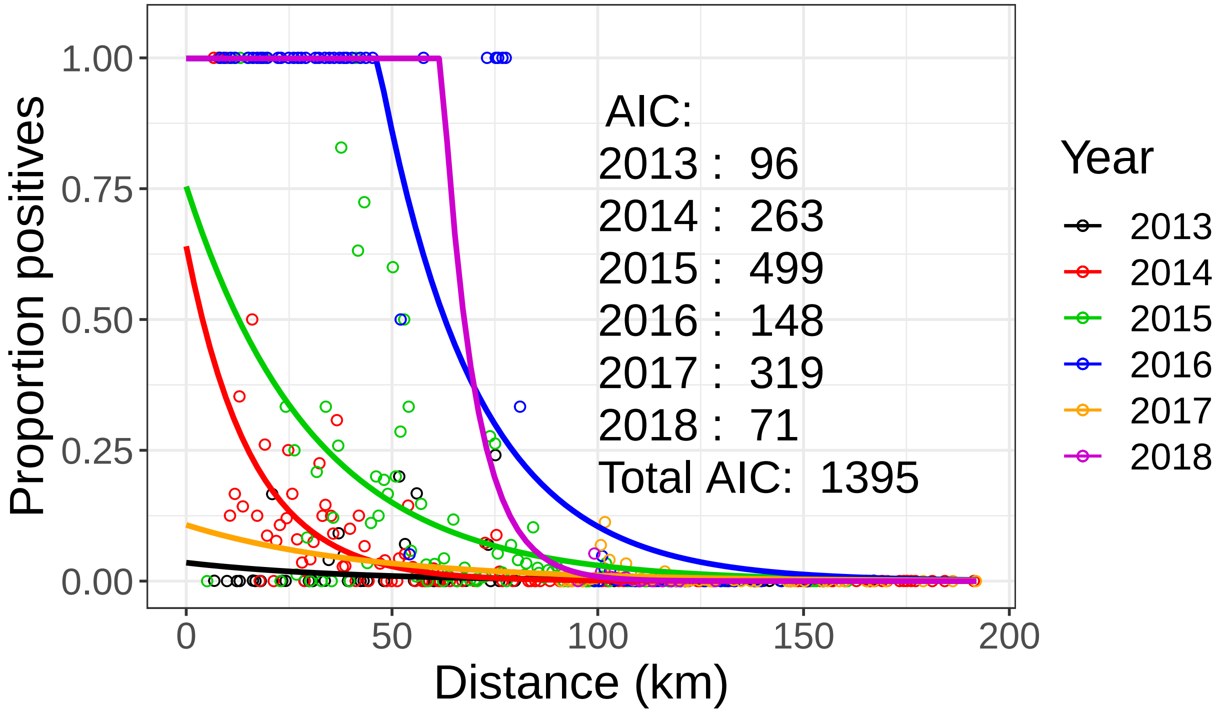

Figure S16. CNE functions fitted on the data with a beta−binomial stochastic distribution. The point of origin is assumed to be Santa Maria di Leuca. X−axis: distance in km (distance circle). Y−axis: Proportion of positive samples.

Rate of Spread

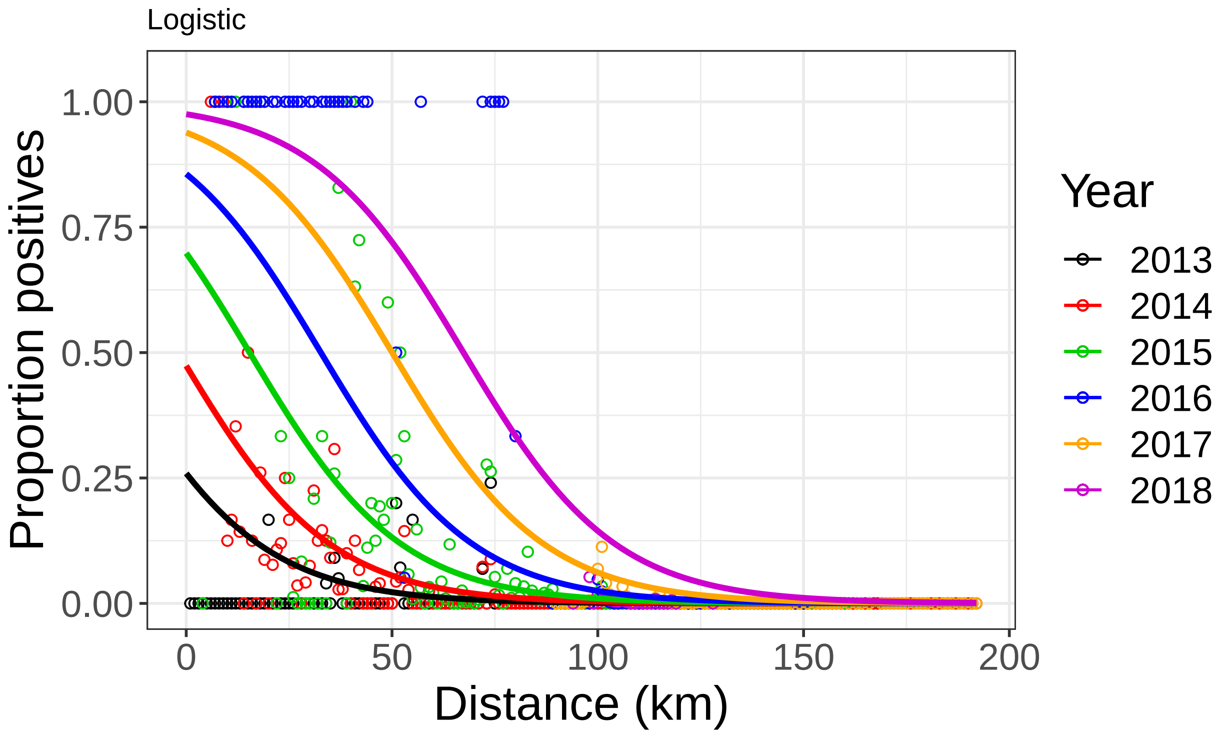

Figure S17. Logistic functions fitted on the data with a beta−binomial stochastic distribution. The point of origin is assumed to be Santa Maria di Leuca. The functions for every year are fit in sequence with a fixed distance between years. X−axis: distance in km (distance circle). Y−axis: Proportion of positive samples.

```
## [1] "Parameter estimations and their lower and upper 95% confidence limits (CLs)"

##              r lower 95% CL upper 95% CL
##     0.05451963   0.04541114   0.06411990

##            x50 lower 95% CL upper 95% CL
##      -19.26236    -30.06379    -10.51853

##              c lower 95% CL upper 95% CL
##       17.32744     14.79717     19.94871

##          theta lower 95% CL upper 95% CL
##       3.706537     2.137537     6.324411
```

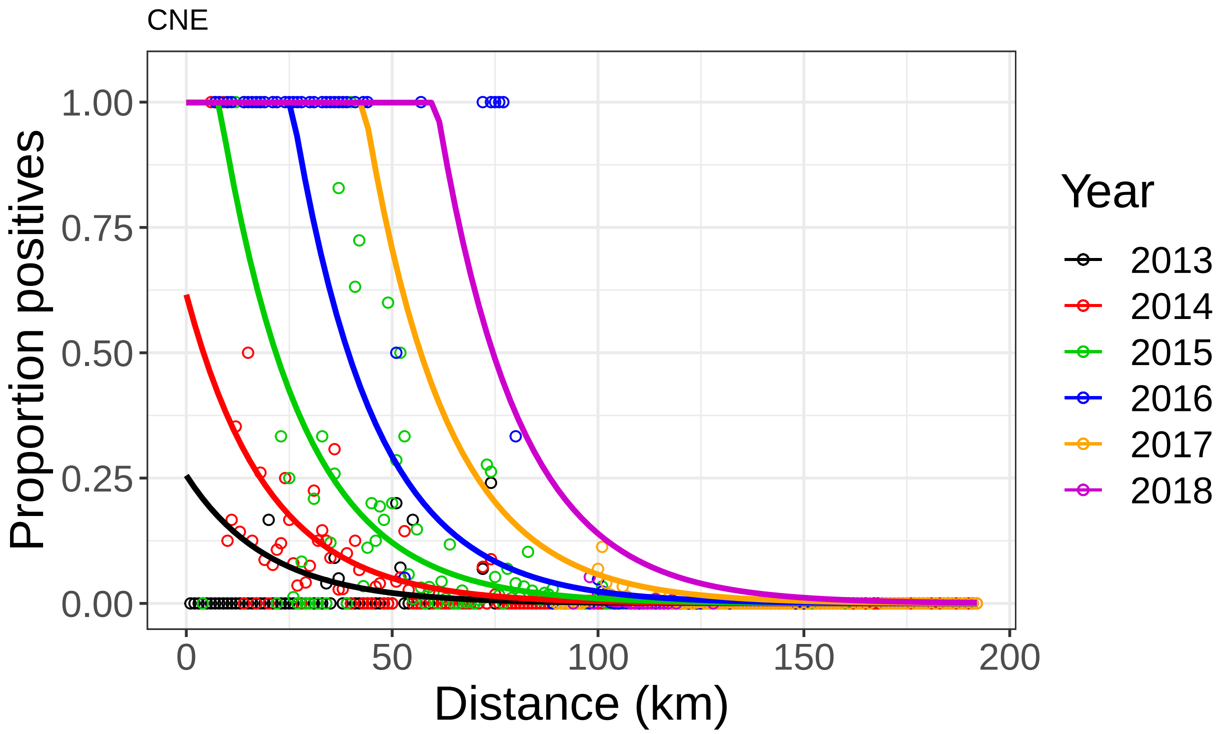

Figure S18. Logistic functions fitted on the data with a beta−binomial stochastic distribution. The point of origin is assumed to be Santa Maria di Leuca. The functions for every year are fit in sequence with a fixed distance between years. X−axis: distance in km (distance circle). Y−axis: Proportion of positive samples.

```
## [1] "Parameter estimations and their lower and upper 95% confidence limits (CLs)"

##              r lower 95% CL upper 95% CL
##     0.05010903   0.03812269   0.05819762

##           x100 lower 95% CL upper 95% CL
##      -27.25179    -44.21774    -19.97723

##              c lower 95% CL upper 95% CL
##       17.58031     14.28047     20.00149

##          theta lower 95% CL upper 95% CL
##       3.908987     2.301327     6.553090
```

2. Otranto

Shape of the Front

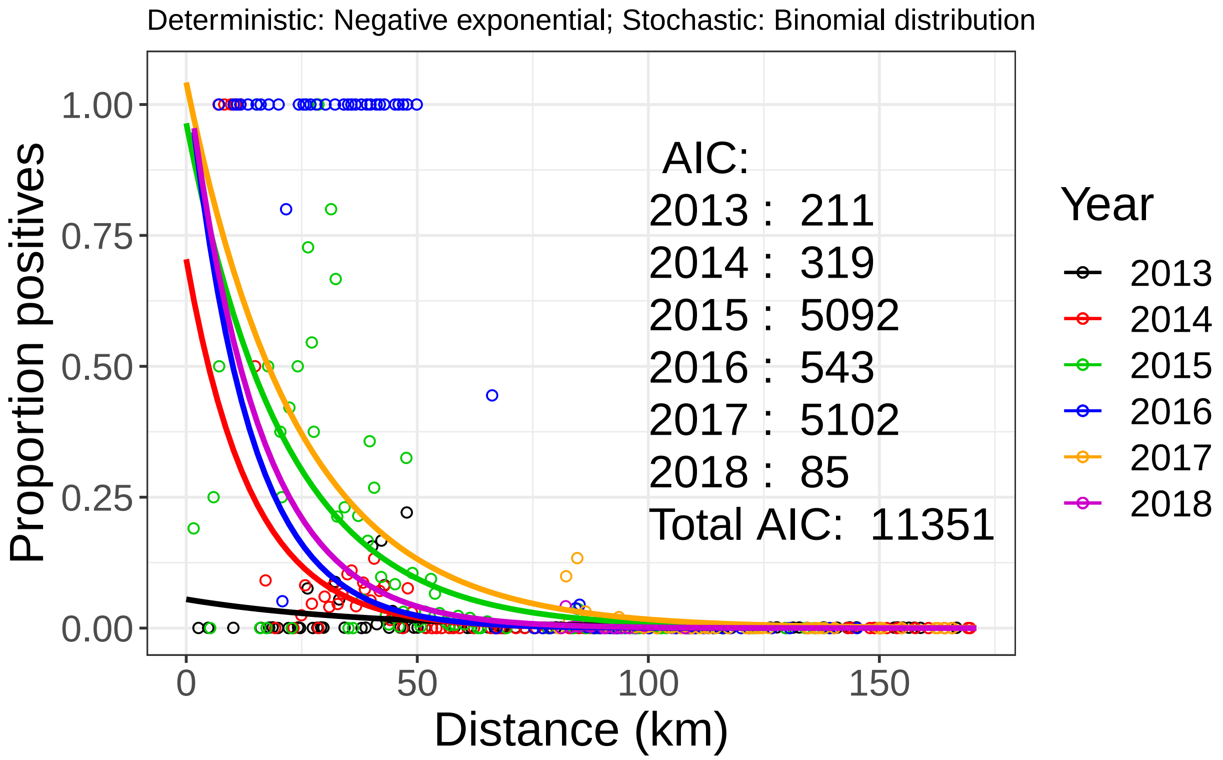

Figure S19. Negative exponential functions fitted on the data with a binomial stochastic distribution. The point of origin is assumed to be Otranto. X−axis: distance in km (distance circle). Y−axis: Proportion of positive samples.

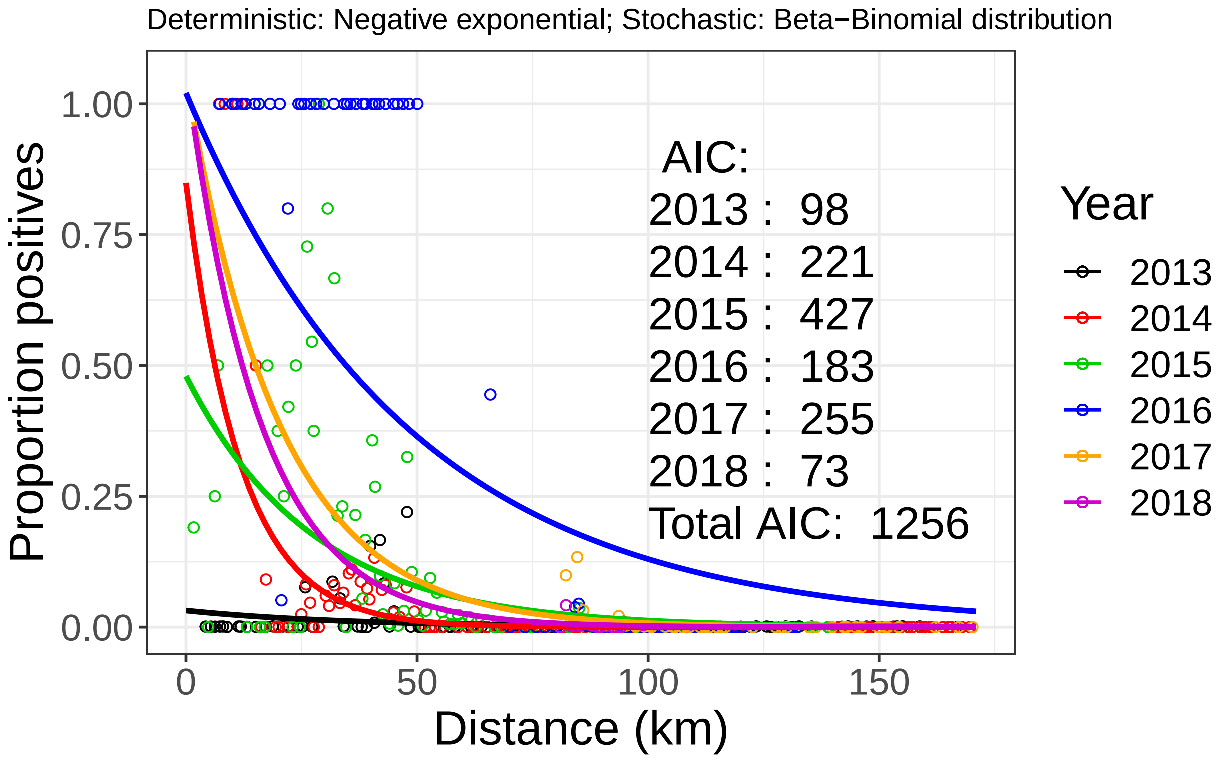

Figure S20. Negative exponential functions fitted on the data with a beta−binomial stochastic distribution. The point of origin is assumed to be Otranto. X−axis: distance in km (distance circle). Y−axis: Proportion of positive samples.

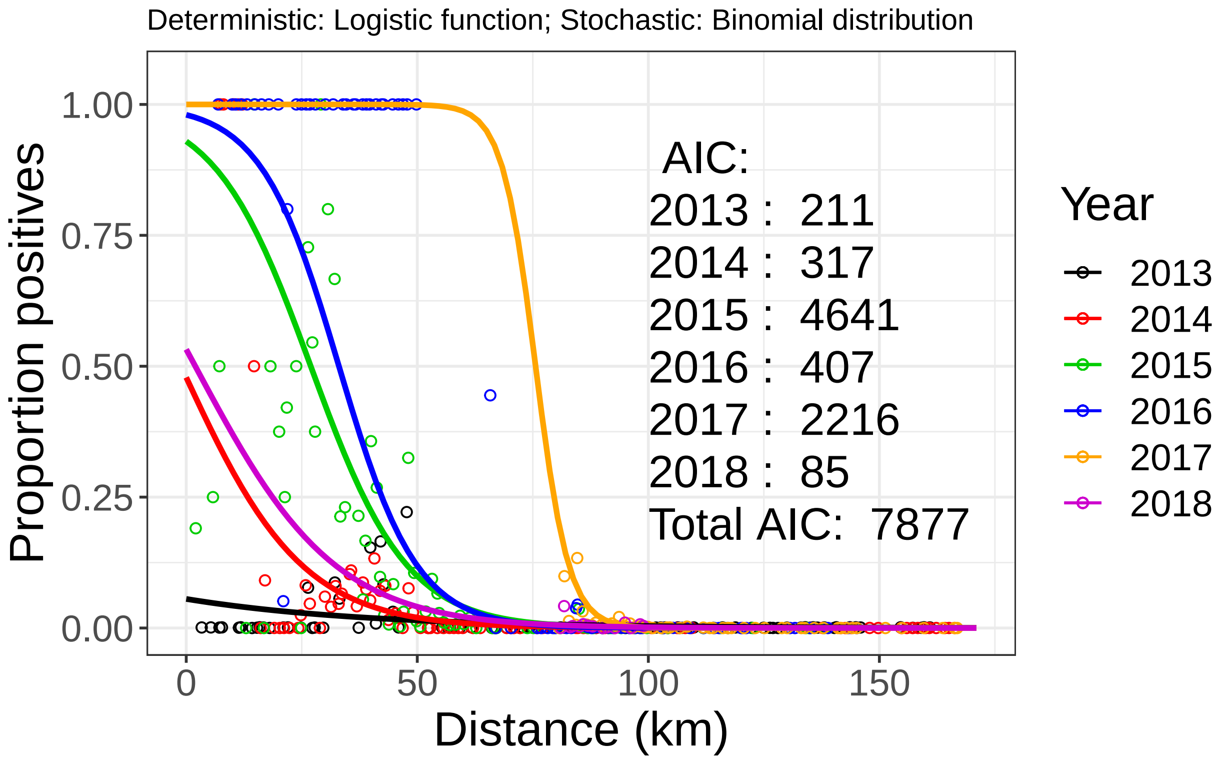

Figure S21. Logistic functions fitted on the data with a binomial stochastic distribution. The point of origin is assumed to be Otranto. X−axis: distance in km (distance circle). Y−axis: Proportion of positive samples.

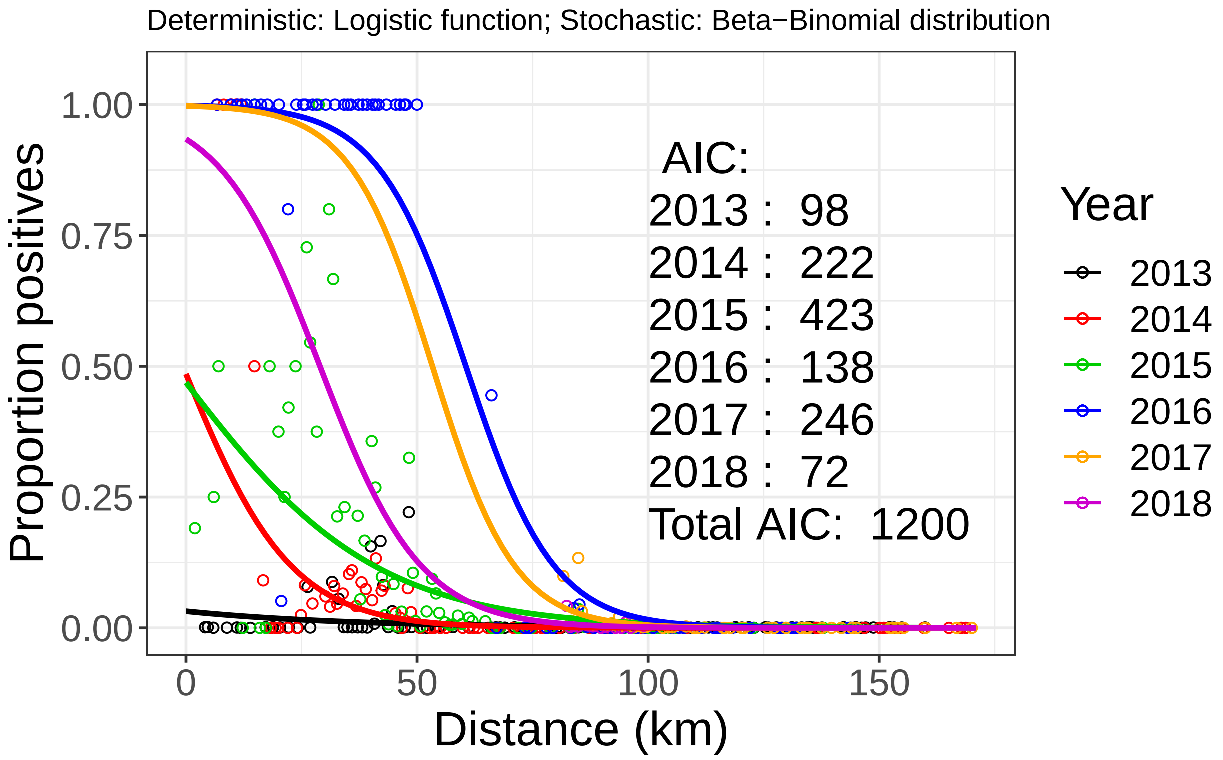

Figure S22. Logistic functions fitted on the data with a beta−binomial stochastic distribution. The point of origin is assumed to be Otranto. X−axis: distance in km (distance circle). Y−axis: Proportion of positive samples.

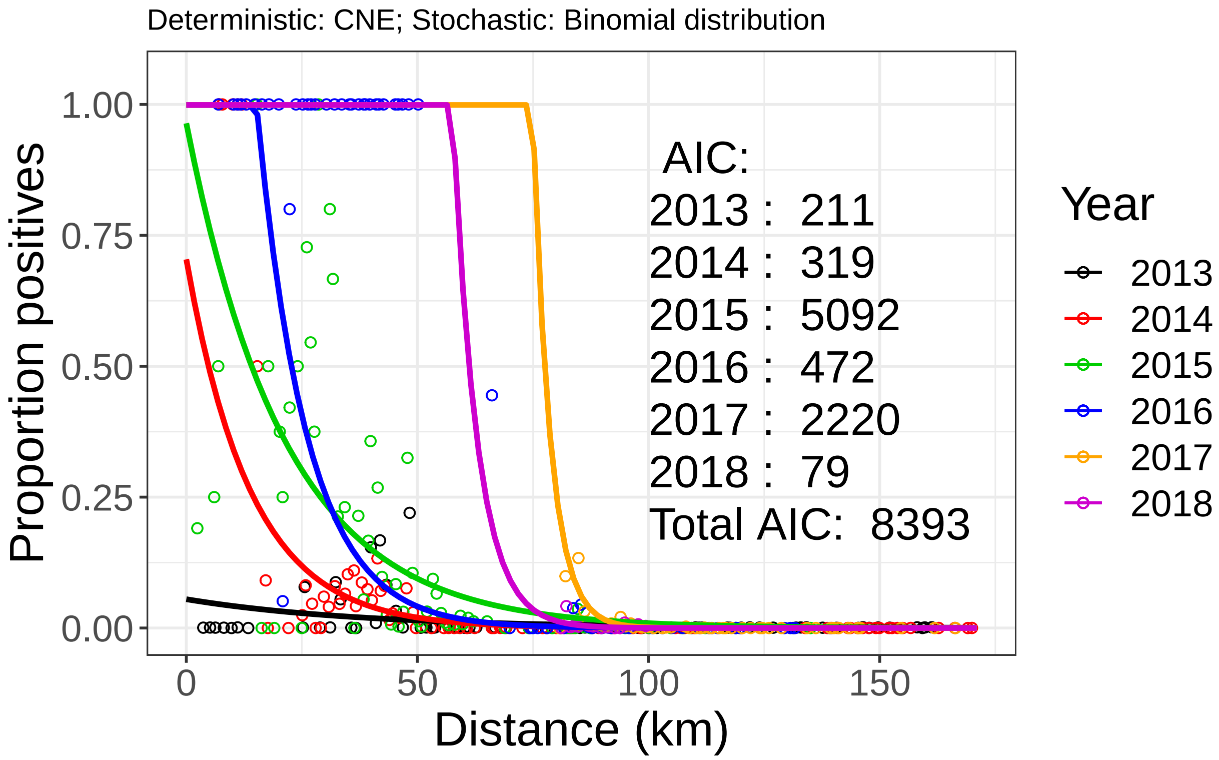

Figure S23. CNE fitted on the data with a binomial stochastic distribution. The point of origin is assumed to be Otranto. X−axis: distance in km (distance circle). Y−axis: Proportion of positive samples.

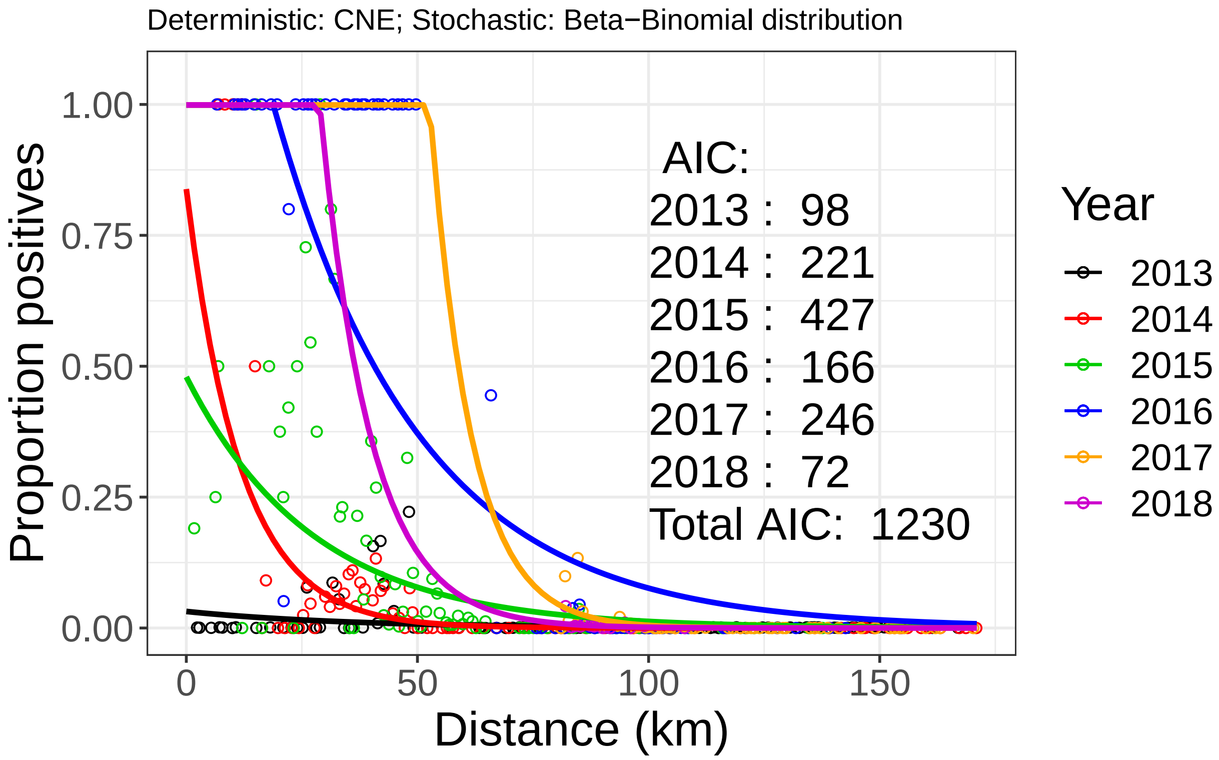

Figure S24. CNE fitted on the data with a beta−binomial stochastic distribution. The point of origin is assumed to be Otranto. X−axis: distance in km (distance circle). Y−axis: Proportion of positive samples.

Rate of Spread

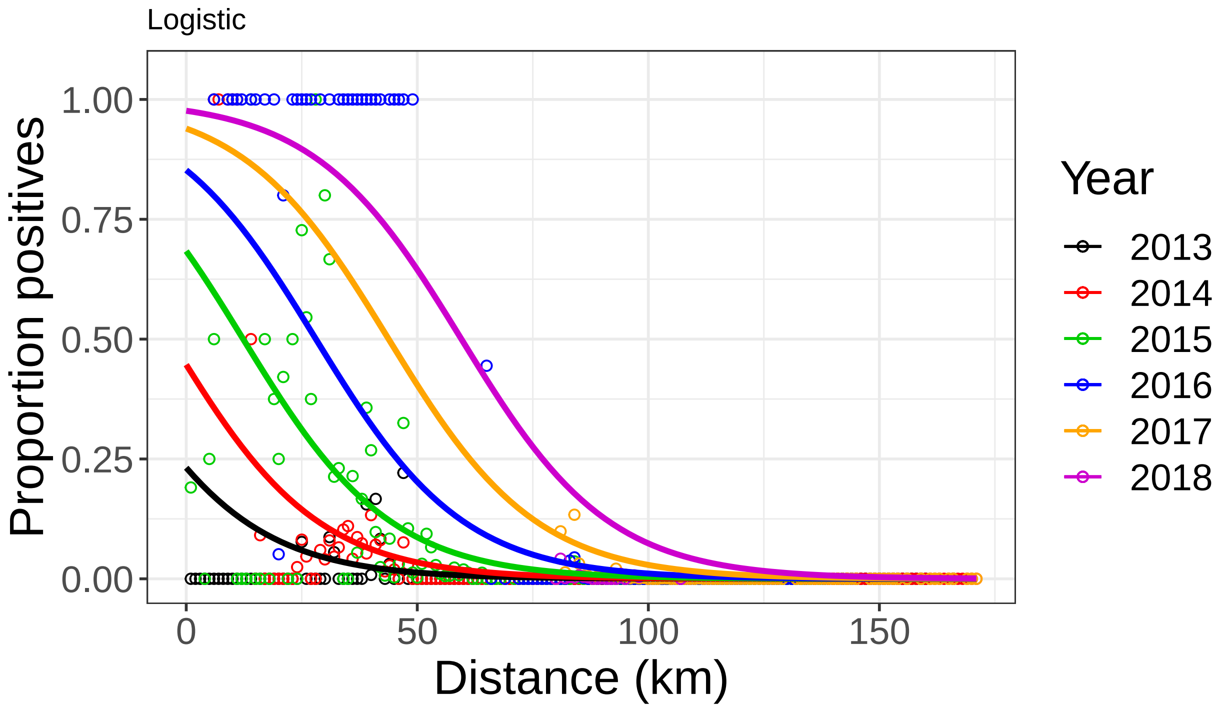

Figure S25. Logistic functions fitted on the data with a beta−binomial stochastic distribution. The point of origin is assumed to be Otranto. It is assumed that the dispersal front retains its shape over time and space and spreads in space at a constant rate. X−axis: distance in km (distance circle). Y−axis: Proportion of positive samples.

```
## [1] "Parameter estimations and their lower and upper 95% confidence limits (CLs)"

##              r lower 95% CL upper 95% CL
##     0.06254527   0.05103663   0.07494243

##            x50 lower 95% CL upper 95% CL
##      -19.16616    -30.46956    -10.00531

##              c lower 95% CL upper 95% CL
##       15.74331     13.29118     18.40251

##          theta lower 95% CL upper 95% CL
##       2.561530     1.408684     4.811686
```

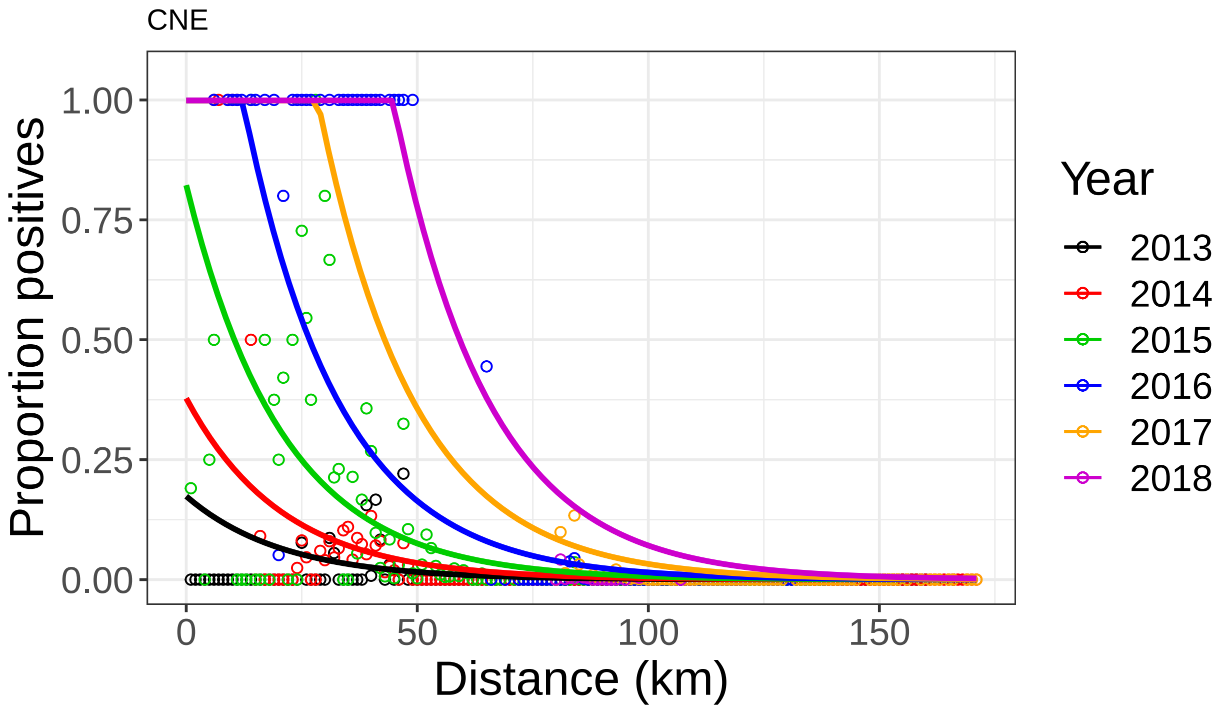

Figure S26. CNE functions fitted on the data with a beta−binomial stochastic distribution. The point of origin is assumed to be Otranto. It is assumed that the dispersal front retains its shape over time and space and spreads in space at a constant rate. X−axis: distance in km (distance circle). Y−axis: Proportion of positive samples.

```
## [1] "Parameter estimations and their lower and upper 95% confidence limits (CLs)"

##              r lower 95% CL upper 95% CL
##     0.04780092   0.04043533   0.05545718

##           x100 lower 95% CL upper 95% CL
##      -36.61094    -46.87982    -28.66980

##              c lower 95% CL upper 95% CL
##       16.26017     13.47316     19.21651

##          theta lower 95% CL upper 95% CL
##       2.617699     1.478713     4.721995
```

3. Maglie

Shape of the Front

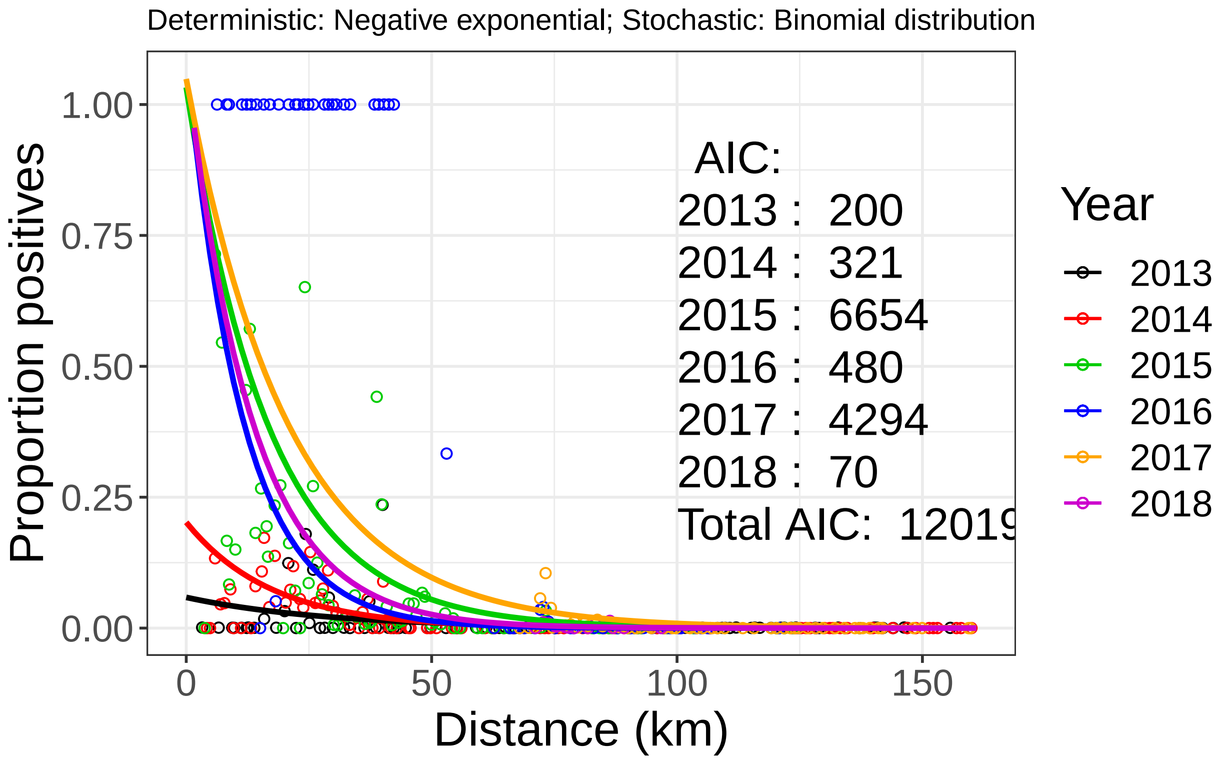

Figure S27. Negative exponential functions fitted on the data with a binomial stochastic distribution. The point of origin is assumed to be Maglie. X−axis: distance in km (distance circle). Y−axis: Proportion of positive samples.

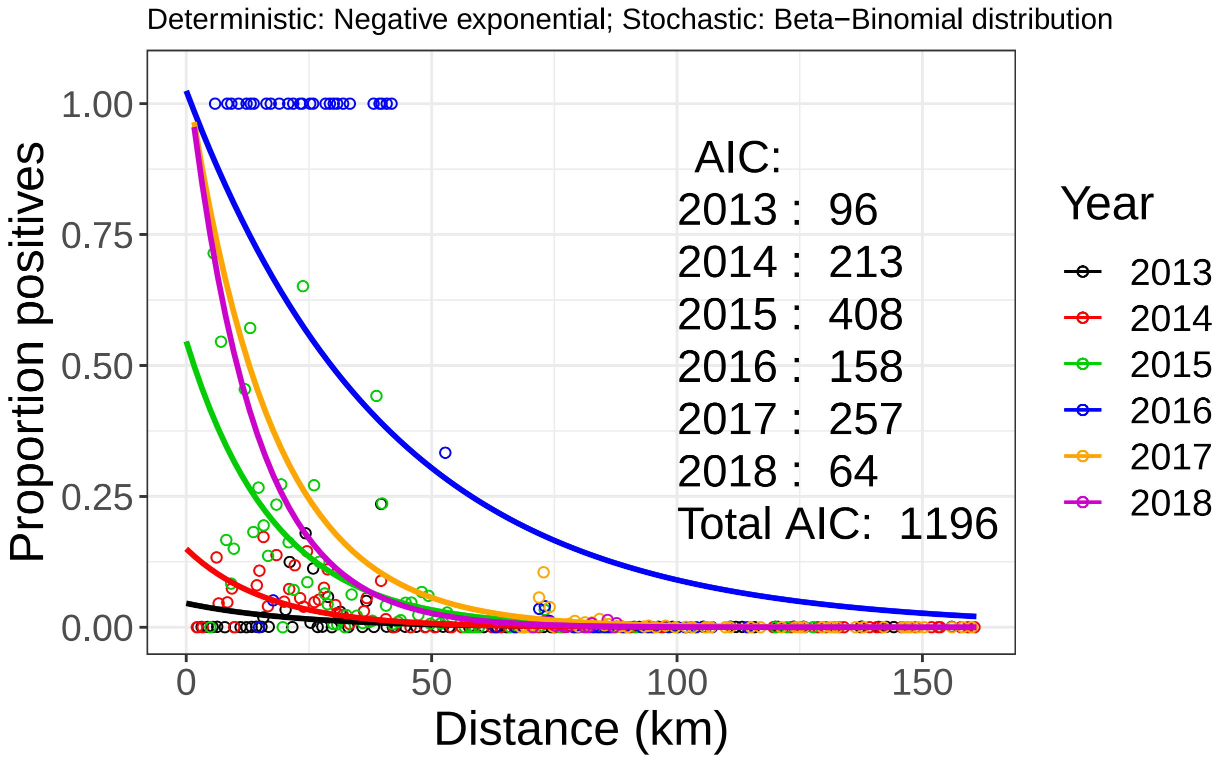

Figure S28. Negative exponential functions fitted on the data with a beta−binomial stochastic distribution. The point of origin is assumed to be Maglie. X−axis: distance in km (distance circle). Y−axis: Proportion of positive samples.

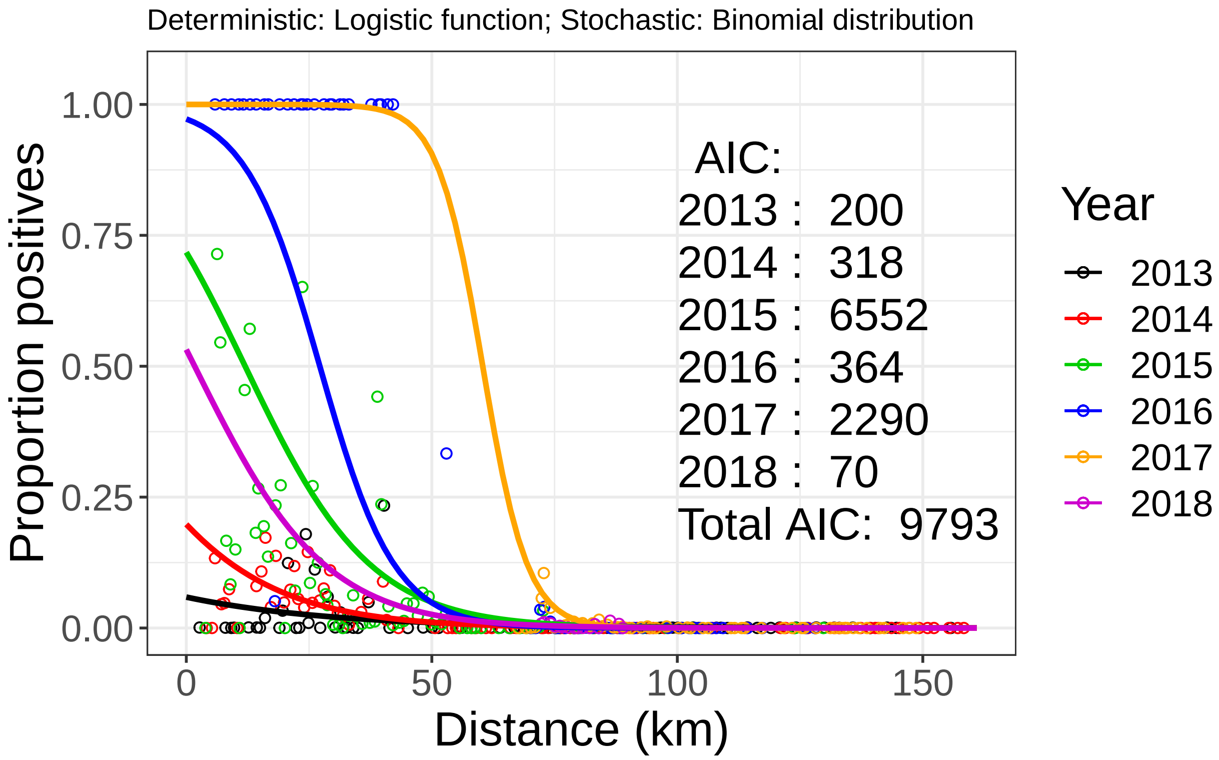

Figure S29. Logistic functions fitted on the data with a binomial stochastic distribution. The point of origin is assumed to be Maglie. X−axis: distance in km (distance circle). Y−axis: Proportion of positive samples.

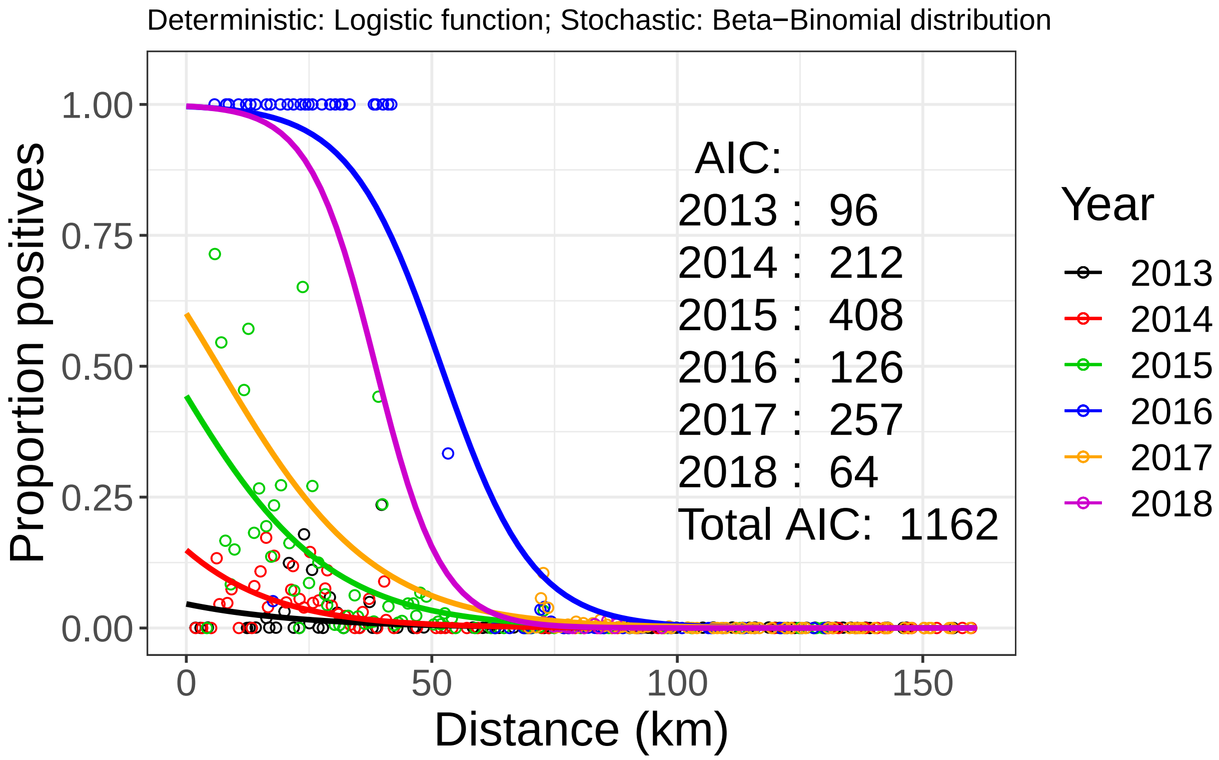

Figure S30. Logistic functions fitted on the data with a beta−binomial stochastic distribution. The point of origin is assumed to be Maglie. X−axis: distance in km (distance circle). Y−axis: Proportion of positive samples.

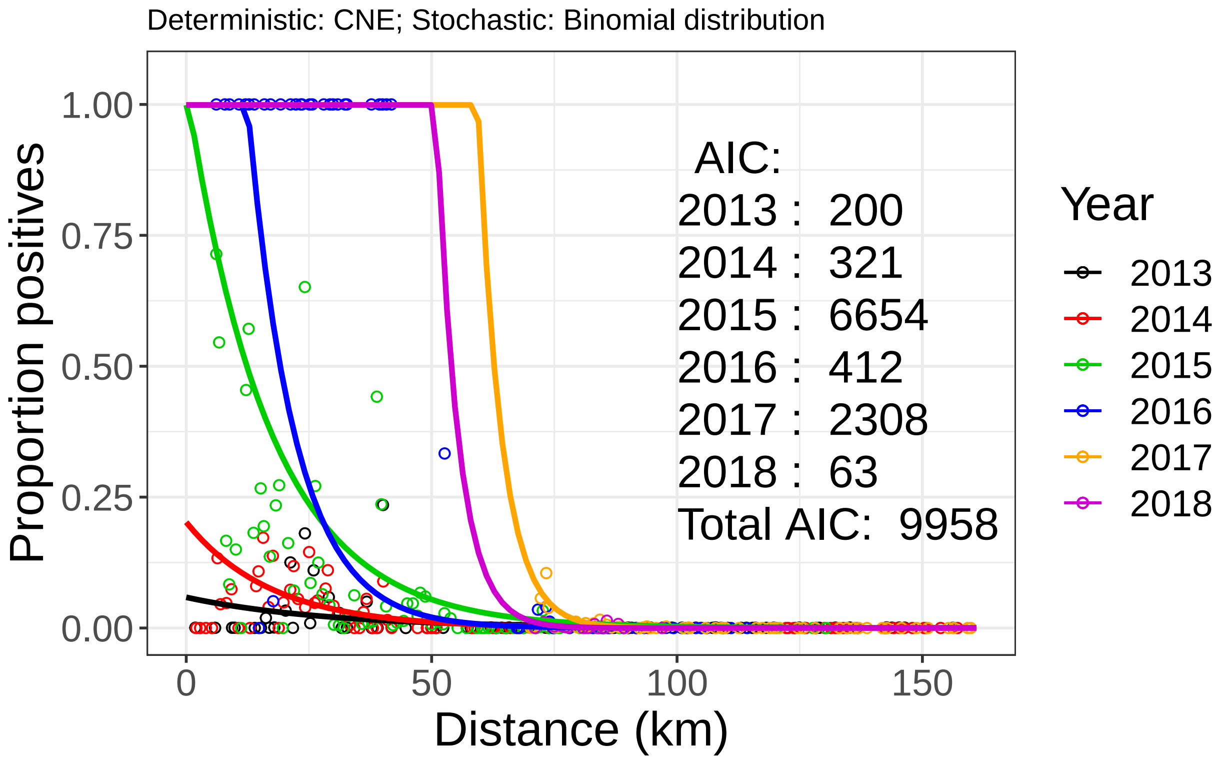

Figure S31. CNE functions fitted on the data with a binomial stochastic distribution. The point of origin is assumed to be Maglie. X−axis: distance in km (distance circle). Y−axis: Proportion of positive samples.

Deterministic: CNE; Stochastic: Beta−Binomial distribution

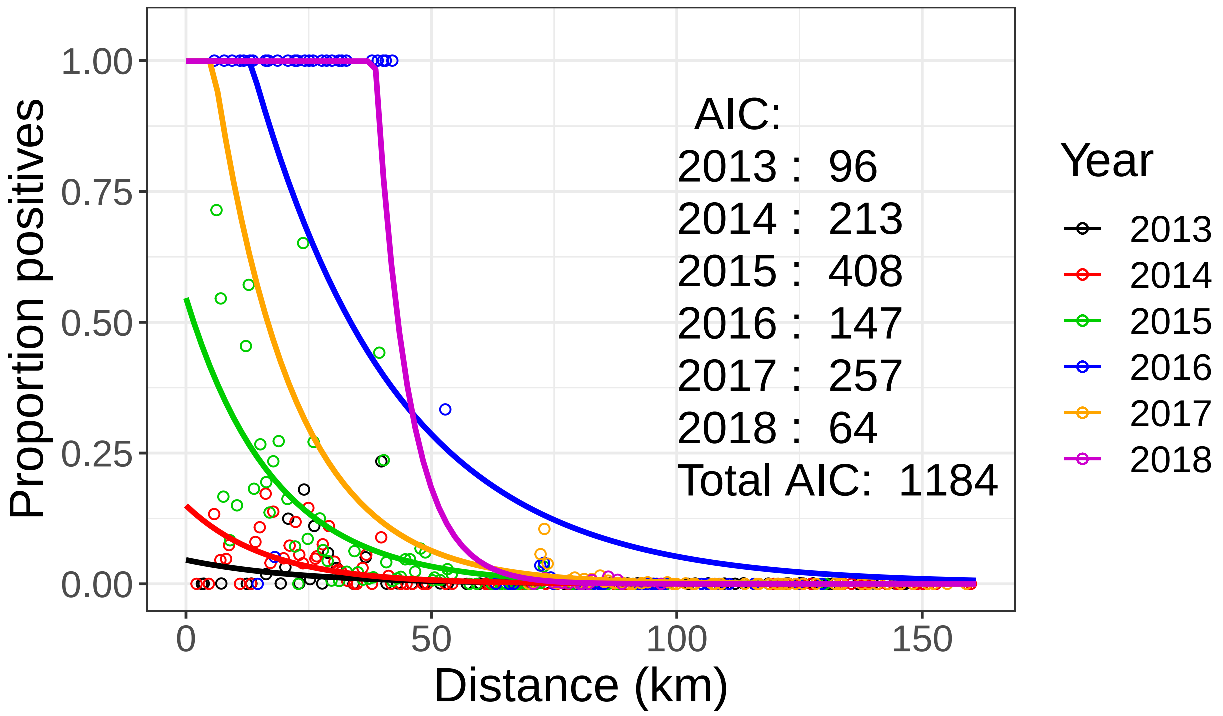

Figure S32. CNE functions fitted on the data with a beta−binomial stochastic distribution. The point of origin is assumed to be Maglie. X−axis: distance in km (distance circle). Y−axis: Proportion of positive samples.

Rate of Spread

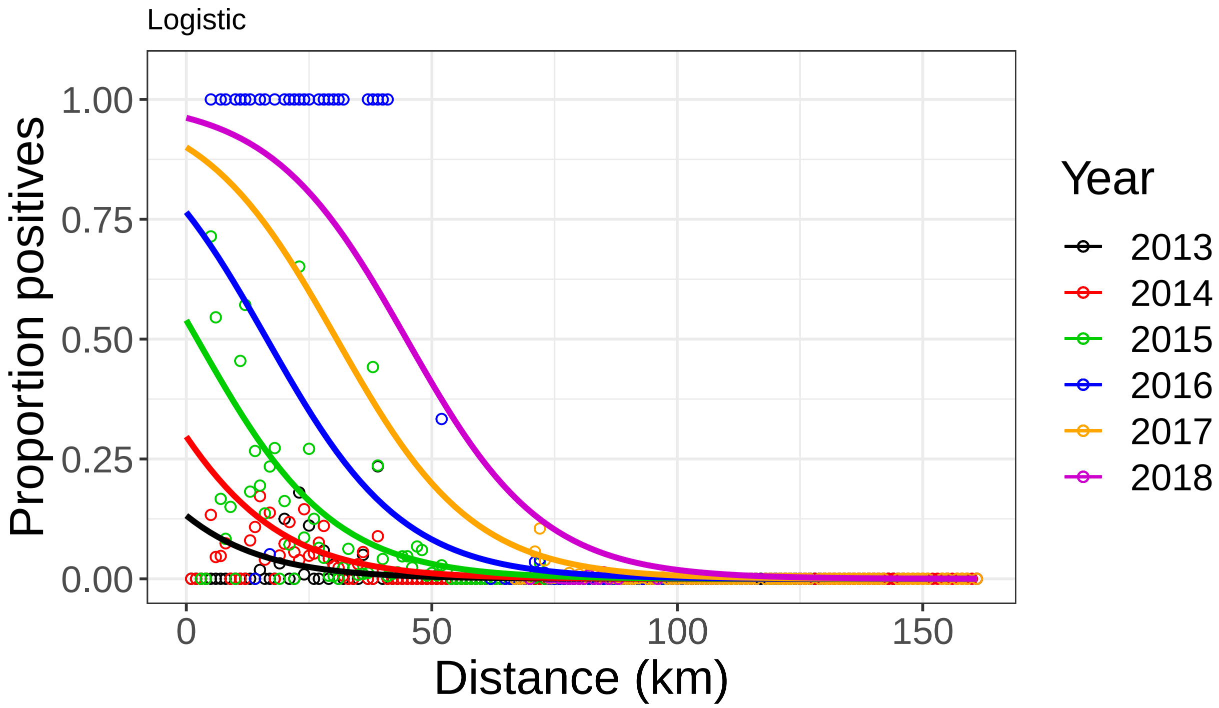

Figure S33. Logistic functions fitted on the data with a beta−binomial stochastic distribution. The point of origin is assumed to be Maglie. It is assumed that the dispersal front retains its shape over time and space and spreads in space at a constant rate. X−axis: distance in km (distance circle). Y−axis: Proportion of positive samples.

```
## [1] "Parameter estimations and their lower and upper 95% confidence limits (CLs)"

##              r lower 95% CL upper 95% CL
##     0.07186582   0.05922972   0.08518933

##            x50 lower 95% CL upper 95% CL
##      -26.22668    -35.43078    -18.79869

##              c lower 95% CL upper 95% CL
##       14.21496     12.10748     16.40629

##          theta lower 95% CL upper 95% CL
##       4.974867     2.616727     9.085707
```

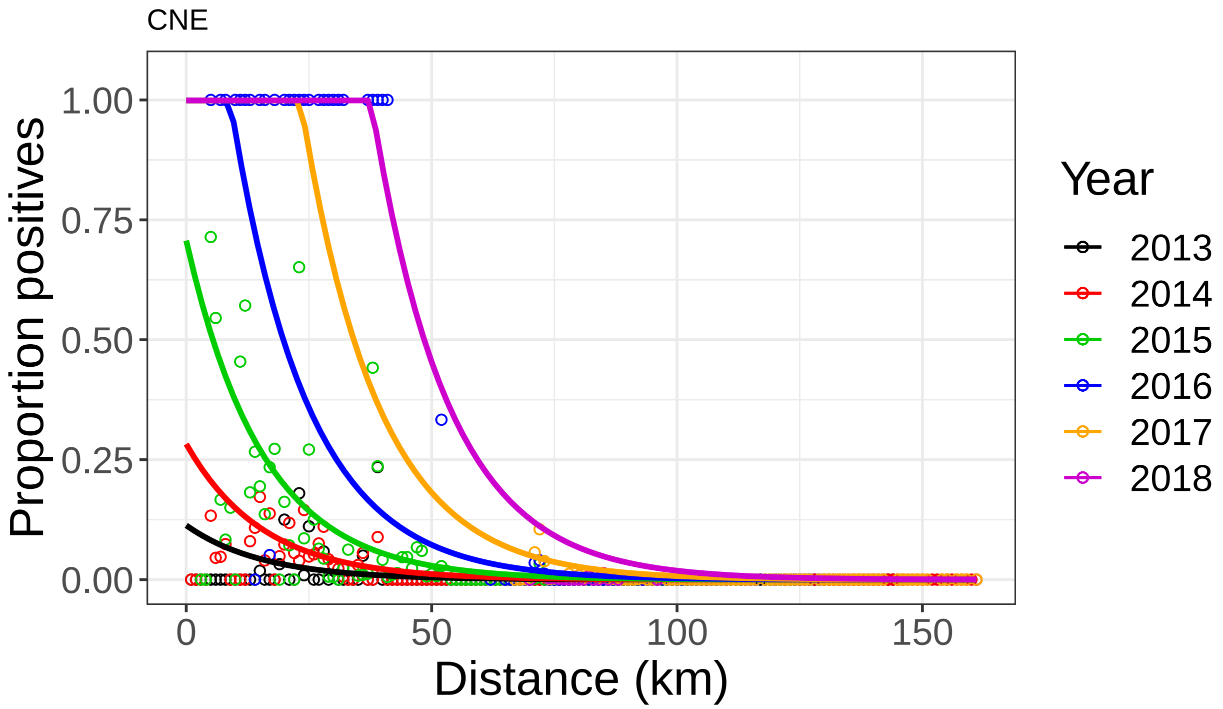

Figure S34. CNE functions fitted on the data with a beta−binomial stochastic distribution. The point of origin is assumed to be Maglie. It is assumed that the dispersal front retains its shape over time and space and spreads in space at a constant rate. X−axis: distance in km (distance circle). Y−axis: Proportion of positive samples.

```
## [1] "Parameter estimations and their lower and upper 95% confidence limits (CLs)"

##              r lower 95% CL upper 95% CL
##     0.06385190   0.05415553   0.07392999

##           x100 lower 95% CL upper 95% CL
##       -34.1414     -41.9702     -27.7966

##              c lower 95% CL upper 95% CL
##       14.35393     12.26515     16.54722

##          theta lower 95% CL upper 95% CL
##       5.451860     2.972650     9.649219
```

Summary of Results

For convenience, the above results are repeated in the Tables S3 - S8. In Tables S6 - S8, the following parameter estimates are given for r, which is the relative growth rate of the disease in the tail in km^{-1}, x, which is the distance in km from the disease origin, Gallipoli, x_{50}, which is the (negative) x-value (distance from Gallipoli) of the half-maximum of the curve at $t = 0$ in km, x_{100}, which is the (negative) x-value where the CNE function curve drops below 1.0 at $t = 0$ in km, t, which is the time since 2013 in years, and the parameter c which is the rate of spread in km per year.

These results show that for all three of the alternative origins the shape of the front is the logistic curve, which is slightly better than the CNE curve. The rate of spread increases for each alternative origin compared to Gallipoli as the origin. This increase in spread can be explained by the fact that the alternative origins are farther away from the direction of the spread (which is north-west, land-inwards). The increase in rate of spread makes up for this increased distance. This is why the increase for Maglie as the origin is less than for Santa Maria di Leuca or Otranto as the origin, since Maglie is less farther away from Gallipoli than the

Table S3. The AIC's for models fitted with Santa Maria di Leuca as origin.

	Santa Maria di Leuca					
	Binomial			Beta-binomial		
Year	Negative exponential	Logistic	CNE	Negative exponential	Logistic	CNE
2013	208	208	208	**96**	**96**	**96**
2014	458	457	458	**263**	**263**	**263**
2015	6024	5972	6024	499	**498**	499
2016	556	312	325	209	**139**	148
2017	4752	3916	9353	**319**	**319**	**319**
2018	75	75	70	99	**71**	**71**
Total	12073	10939	11038	1485	**1384**	1395

Table S4. The AIC's for models fitted with Otranto as origin.

	Otranto					
	Binomial			Beta-binomial		
Year	Negative exponential	Logistic	CNE	Negative exponential	Logistic	CNE
2013	211	211	211	**98**	**98**	**98**
2014	319	317	319	**221**	222	**221**
2015	5092	4641	5092	427	**423**	427
2016	543	407	472	183	**138**	166
2017	5102	2216	2220	255	**246**	**246**
2018	85	85	79	73	**72**	**72**
Total	11351	7877	8393	1256	**1200**	1230

Table S5. The AIC's for models fitted with Maglie as origin.

	Maglie					
	Binomial			Beta-binomial		
Year	Negative exponential	Logistic	CNE	Negative exponential	Logistic	CNE
2013	200	200	200	**96**	**96**	**96**
2014	321	318	321	213	**212**	213
2015	6654	6552	6654	**408**	**408**	**408**
2016	480	364	412	158	**126**	147
2017	4294	2290	2308	**257**	**257**	**257**
2018	70	70	63	**64**	**64**	**64**
Total	12019	9793	9958	1196	**1162**	1184

Table S6. The results of the parameter estimations with Santa Maria di Leuca as origin. The values between the brackets are the 95% confidence limits (CL's)

	Santa Maria di Leuca	
Parameter	Logistic	CNE
r	0.055 (0.045, 0.064)	0.050 (0.038, 0.058)
x_{50}/x_{100}	-19.26 (-30.06, -10.52)	-27.25 (-44.22, -19.98)
c	17.33 (14.80, 19.95)	17.58 (14.28, 20.00)
theta	3.71 (2.14, 6.32)	3.91 (2.30, 6.55)

other two. The increase in the rate of spread is substantial (4 - 7 km per year), indicating the importance of choosing the correct point of origin.

Table S7. The results of the parameter estimations with Otranto as origin. The values between the brackets are the 95% CL's

	Otranto	
Parameter	Logistic	CNE
r	0.063 (0.051, 0.075)	0.048 (0.040, 0.055)
x_{50}/x_{100}	-19.16 (-30.47, -10.01)	-36.61 (-46.88, -28.67)
c	15.74 (13.29, 18.40)	16.26 (13.47, 19.22)
theta	2.56 (1.41, 4.81)	2.62 (1.48, 4.72)

Table S8. The results of the parameter estimations with Maglie as origin. The values between the brackets are the 95% CL's

	Maglie	
Parameter	Logistic	CNE
r	0.072 (0.059, 0.085)	0.064 (0.054, 0.074)
x_{50}/x_{100}	-26.23 (-35.43, -18.80)	-34.14 (-41.97, -27.80)
c	14.21 (12.11, 16.41)	14.35 (12.27, 16.55)
theta	4.97 (2.62, 9.09)	5.45 (2.97, 9.65)
