# Supplementary material for: Shape and rate of movement of the invasion front of Xylella fastidiosa spp. pauca in Puglia
Source: Sci Rep. 2021 Jan 13;11:1061. doi: 10.1038/s41598-020-79279-x (PMC7806996; doi:10.1038/s41598-020-79279-x)
Supplement: Supplementary file 3 — Supplementary Information 3. [file 41598_2020_79279_MOESM3_ESM.pdf]

Shape and rate of spread of the invasion front of *Xylella fastidiosa* spp. *pauca* in Puglia

Supplementary Analysis 3: Simulation of data starting from alternative disease origins that are analysed with Gallipoli as assumed place of origin

David Kottelenberg Lia Hemerik Maria Saponari Wopke van der Werf

Analysis with different simulation origins

In this analysis we perform simulations of disease spread for three origins different from Gallipoli, and estimate the rate of spread with Gallipoli as the assumed origin. We simulate a data set with disease prevalence in Puglia. The spread of this simulated disease has a front with an associated range expansion rate. The point of origin for this disease simulation is one of the following:

- Santa Maria di Leuca (longitude: 18.359826; latitude: 39.796772)
- Otranto (longitude: 18.490599; latitude: 40.146590)
- Maglie (longitude: 18.299162; latitude: 40.121722)

Using these simulated datasets, we calculate the rate of spread when analysing the disease occurrence while assuming Gallipoli is the point of origin (which in this case it is not). This way, we can see the impact of choosing Gallipoli as the point of origin when the disease has a different point of origin. From this analysis we calculate the mean bias and root-mean-squared error (RMSE) of the rate of spread estimations. The code used for the analyses of these origins is the same as the code found in *Supplementary analysis 3.R* on Github (https://github.com/DBKottelenberg/OQDS_Xf_Puglia), which is similar to *Sample Simulation - Puglia Data.R* except for the coordinates used, which is the coordinates as above for every different origin, and the range of rate of spread used, which is 8 to 20 km/year instead of 5 to 16 km/year because of the higher estimated rate of spread for these origins (see Supplementary Analysis 2). Also, we calculate disease prevalence on the spatially explicit sampled points of the original data.

We only show the results for the logistic curve as input function and analysis function, because this is the best fitting shape of the front for every origin (see Supplementary Analysis 2). The full results of the simulations can be found on Github in the files *dat_accuracy_SMdL.xlsx* (Santa Maria di Leuca), *dat_accuracy_O.xlsx* (Otranto), and *dat_accuracy_M.xlsx* (Maglie).

Results

The summarized results are displayed in Table S9.

Table S9. The mean Bias and root-mean-squared error (RMSE) of the estimated rate of spread of the simulated data with different origins.

Origin	Mean.Bias	RMSE
Santa Maria di Leuca	-6.73	7.76
Otranto	-4.16	7.16
Maglie	-2.36	2.64

These results show that choosing Gallipoli as the point of origin in the analysis of simulated data with different points of origin generally gives an underestimation of the rate of spread. This can be explained by the fact that Gallipoli lies North-West from the points of origin, which is the direction the disease spreads. This means that the distance the disease has spread over time is less when measured from Gallipoli then when measured from the actual (simulated) point of origin. For low true rates of spread, the estimated rate of spread can even be negative, since the disease would come closer to Gallipoli over the years (see the full results in the Github files). Additionally, we see that the RMSE is very high for all three points of origin, indicating a high uncertainty of the calculated bias. In conclusion, if the point of origin of Olive Quick Decline Syndrome is Santa Maria di Leuca, Otranto, or Maglie, analysing the rate of spread with an assumed point of origin in Gallipoli yields inaccurate results with possibly unrealistically low estimations.
